# Automated Classification of Sleep–Wake States and Seizures in Mice

**DOI:** 10.1523/ENEURO.0226-25.2025

**Published:** 2025-10-23

**Authors:** Brandon J. Harvey, Viktor J. Olah, Lauren M. Aiani, Lucie I. Rosenberg, Danny J. Lasky, Benjamin Moxon, Nigel P. Pedersen

**Affiliations:** ^1^Graduate Program in Neuroscience, Emory University, Atlanta, Georgia 30322; ^2^Department of Neurology, University of California, Davis, Davis, California 95618; ^3^Department of Cell Biology, Emory University, Atlanta, Georgia 30322; ^4^Department of Genetics, Emory University School of Medicine, Atlanta, Georgia 30322; ^5^Graduate Program in Neuroscience, University of California, Davis, Davis, California 95618; ^6^Department of Neurological Surgery, University of California, Davis, Davis, California 95618; ^7^Center for Neuroengineering and Medicine, University of California, Davis, Davis, California 95618

**Keywords:** epilepsy, machine learning, seizures, sleep, sleep–wake

## Abstract

Sleep–wake states bidirectionally interact with epilepsy and seizures, but the mechanisms are unknown. A barrier to comprehensive characterization and the study of mechanisms has been the difficulty of annotating large chronic recording datasets. To overcome this barrier, we sought to develop an automated method of classifying sleep–wake states, seizures, and the postictal state in mice ranging from controls to mice with severe epilepsy with accompanying background electroencephalographic (EEG) abnormalities. We utilized a large dataset of recordings, including electromyogram, EEG, and hippocampal local field potentials, from control and intra-amygdala kainic acid-treated mice. We found that an existing sleep–wake classifier performed poorly, even after retraining. A support vector machine, relying on typically used scoring parameters, also performed below our benchmark. We then trained and evaluated several multilayer neural network architectures and found that a bidirectional long short-term memory–based model performed best. This “Sleep–Wake and Ictal State Classifier” (SWISC) showed high agreement between ground-truth and classifier scores for all sleep and seizure states in an unseen and unlearned epileptic dataset (average agreement 96.41% ± SD 3.80%) and saline animals (97.77 ± 1.40%). Channel dropping showed that SWISC was primarily dependent on hippocampal signals yet still maintained good performance (∼90% agreement) with EEG alone, thereby expanding the classifier's applicability to other epilepsy datasets. SWISC enables the efficient combined scoring of sleep–wake and seizure states in mouse models of epilepsy and healthy controls, facilitating comprehensive and mechanistic studies of sleep–wake and biological rhythms in epilepsy.

## Significance Statement

We describe a unique machine learning classifier that can identify sleep–wake states and seizures from continuous electroencephalographic (EEG) signals in mice with varying degrees of epilepsy-related EEG abnormalities. This new tool will be necessary for the epilepsy research community, facilitating and replacing laborious human scoring of long recordings and large groups of mice.

## Introduction

While the relationship between sleep and seizures has been widely appreciated for centuries (Janz, 1962; Shouse and Sterman, 1982; Crespel et al., 1998), mechanisms remain obscure. As a prelude to rodent studies examining this relationship, we sought a means to comprehensively label (score) sleep, wake, and seizure-related activity in large datasets from continuous chronic mouse electroencephalographic (EEG) recordings.

Several factors hinder the meticulous investigation of sleep in rodent models of epilepsy: Firstly, there are variable changes in the EEG background and prominent interictal EEG abnormalities (Pitkänen et al., 2017). Secondly, these abnormalities are not static, typically evolving from seizure induction throughout the recording period. Lastly, there is substantial variability in the number, severity (Almeida Silva et al., 2016), and electrophysiological morphology (Henshall et al., 2000) of seizures and interictal findings, necessitating larger cohorts than might be needed for studies of sleep–wake alone. These large datasets often involve thousands of hours of multichannel recording. Manual scoring of this data for both seizures and sleep–wake is often impractical; a trained expert scorer may take >25 min to score sleep–wake for 12 h of data (Kloefkorn et al., 2020) and even longer when EEGs are abnormal.

Sleep–wake and seizure classification have independently been achieved. Earlier approaches to sleep classification, using linear discrimination, depend on highly simplified features such as assessing the EEG power ratio in the theta and delta bands and mean and standard deviation of electromyogram (EMG; Costa-Miserachs et al., 2003). Overall, these existing methods likely depend on typical EEG background rhythms and features that are disrupted in epilepsy (Kilias et al., 2018; Song et al., 2024). More complex methodologies for sleep scoring include approaches utilizing support vector machines (SVMs; Lampert et al., 2015) and machine learning-based approaches. The latter type of algorithms includes several classifiers driven by convolutional feature extraction. AccuSleep (Barger et al., 2019) uses a linear classification layer, and MC-SleepNet (Yamabe et al., 2019) uses a bidirectional long short-term memory layer (BiLSTM) with a dense layer for classification. Previously, techniques including convolutional feature extraction were used to score sleep and cataplexy in cataplexic mice (Exarchos et al., 2020). However, these existing sleep classifiers are trained on nonepileptic mice with a normal EEG background and do not account for the seizures and the postictal state.

Several effective seizure detection approaches have been published. Methods include parametric (Tieng et al., 2017), machine learning (Wei et al., 2020), and deep learning-based (Jang and Cho, 2019) algorithms. These previous works are also adequate for identifying seizures across various mouse models of epilepsy (Wei et al., 2021). However, like the existing published sleep scoring classifiers, none combine sleep–wake and seizure classification.

Large datasets with prolonged recording are needed to study the important phenomenological and mechanistic relationships between sleep–wake and biological rhythms. These datasets necessitate an automated way to perform combined sleep–wake and epilepsy-related classification. We aimed to create an automated sleep–wake and seizure scoring method that could batch-process and accurately score files from control mice and mice with varying degrees of epilepsy-related EEG background abnormalities. We also sought to use our sleep–wake and seizure state data to evaluate and directly compare which signal features and approaches resulted in the most accurate sleep and seizure identification. We focused on machine learning methods that are either theoretically appropriate or empirically suited to classifying EEG time series data. As a benchmark, we sought to achieve a classification accuracy comparable to that seen with human scoring, as determined by inter-rater agreement. The inter-rater accuracy for sleep scoring in mice between scorers in our laboratory, defined as the percentage of epochs where all scorers agree on a label, is high at >93% (Kloefkorn et al., 2020), in accordance with other reports of 92% (Rytkönen et al., 2011). Here, we describe a highly accurate method for simultaneous automated sleep–wake and seizure classification, the Sleep–Wake and Ictal State Classifier (SWISC).

## Materials and Methods

### Mice

Mice (9–31 weeks old; *n* = 79) of either sex (*n* = 34 male; *n* = 45 female) were used in accordance with the Emory University Institutional Animal Care and Use Committee. Mice in this dataset were obtained from Jackson Laboratory and were wild-type C57BL/6J (*n* = 16; *n* = 8 of each sex, Stock Number 000664) or VGAT-ires-Cre Knock-In C57BL/6J (VGAT-Cre; *n* = 63; *n* = 37 female and *n* = 26 male; Stock Number 028862). VGAT-Cre mice were used to obtain baseline sleep–wake and epilepsy data as a prelude to future studies with this genotype. The intra-amygdala kainic acid (IAKA) model was used for C57BL/6J mice given a relatively lower mortality rate compared with other chemical kindling approaches in this strain (Conte et al., 2020), as well as its utility for later electrical kindling experiments (Straub et al., 2020). All animals were bred in our animal facility with the oversight of the Department of Animal Resources. Breeding procedures included backcrossing every five generations. DNA samples were obtained via ear punch before surgery to determine the genotype using polymerase chain reaction per the Jackson Laboratory protocol. Mice were provided with food and water *ad libitum* and maintained on a 12 h light/dark cycle (lights on 7 A.M.–7 P.M.). Cages were changed weekly and in the same session for all mice. Mortality rates were 20% (*n* = 13 of *n* = 63) for VGAT-Cre mice and 18.75% (*n* = 3 of *n* = 16) wild types.

Exclusion criteria included death between the beginning of baseline recording and 4 d post-IAKA administration, technical failure before the study endpoint 3 weeks after IAKA administration, or membership in the first experimental cohort with a guide cannula.

### Surgery

Surgical procedures have been described previously (Zhu et al., 2020). Briefly, mice were induced with ketamine (100 mg/kg, i.p.) and xylazine (10 mg/kg, i.p.), followed by meloxicam (5 mg/kg, s.c.) in 1 cc saline, and anesthesia was maintained with isoflurane (0–1.5%). Four holes were drilled for head plate screws, two for depth electrodes, and one for a guide cannula targeting the basolateral amygdala. Bilateral hippocampal depth electrodes were placed in the perforant path region, immediately overlying the dorsal blade of the dentate gyrus (±2.00 mm ML, −2.53 mm AP, −1.80 mm DV from the brain surface). Using a headplate-mounted recording montage developed by our laboratory, screw electrodes for electrocorticography (ECoG) were placed in the left frontal (−1.30 mm ML, +1.00 mm AP) and right parietal bones (+2.80 mm ML, −1.50 mm AP), as well as a reference over the midline cerebellum (0 mm ML, −6.00 mm AP) and a ground in the right frontal bone (+1.30 mm ML, +1.00 mm AP). Finally, the guide cannula (5 mm, Plastics1) was implanted with the tip 1.75 mm dorsal to the basolateral amygdala target (−2.75 or −3.25 mm ML, −0.94 mm AP, −3.80 mm DV from the brain surface). EMG paddles (Plastics1) were inserted subcutaneously above the posterior neck muscles, and instrumentation was then secured with cyanoacrylate adhesive and dental cement. The mice were then allowed time to recover from anesthesia and regain their righting reflex before being singly housed in 7-inch-diameter clear acrylic recording barrels with food and water available *ad libitum*, nesting material, and a 12 h light/dark cycle.

### Mouse recording

Mice recovered from surgery for 3–4 d and were then connected to the tether for 2 d of habituation before 7 d of baseline recording. Video (recorded at 7 fps), EEG [ECoG and bilateral hippocampal field potential (HPC-L/R)], and EMG were recorded at a sampling rate of 2 kHz continuously throughout the experiment, without online filtering. A 1 MP day–night camera was used for video recording (ELP). Preamplifying headsets (8406-SE31M, 100× gain, Pinnacle Technologies) were used with a commutator (model 8408, Pinnacle Technologies) and analog breakout boxes (8443-PWR, Pinnacle Technologies) with additional gain and digitization [Power 1401, Cambridge Electronic Design (CED)]. Synchronized video and EEG/EMG files were saved every 12 h and automatically restarted with Spike2 (v9.10, CED).

### Kainic acid injection

After continuous baseline recording for 7 d, either kainic acid (IAKA; *n* = 51) or normal saline vehicle (*n* = 7) was injected into the basolateral amygdala 5–7 h after lights on (0.3 μg in 200 nl over 5 min) via the internal cannula after removal of the stylet. Video-EEG recordings were continued throughout the IAKA infusion. Mice were injected with diazepam (5 mg/kg, i.p.) to terminate status epilepticus 40 min after the end of the IAKA infusion. The recording then continued for an additional 3 weeks.

### Manual sleep and seizure scoring

Sleep and seizure scoring was performed by one of three authors (L.M.A., L.I.R., N.P.P.) and then confirmed by those with the most experience (L.M.A., N.P.P.). All files were therefore scored by at least two different trained experts. Sleep scoring was performed manually by a conventional approach with visual assessment of the four recording electrographic channels, a spectrogram of EEG and hippocampal depth electrode, root-mean-squared (RMS) EEG, with video recording available for disambiguation (not used for scoring, but available for Racine staging).

**Figure 1.**
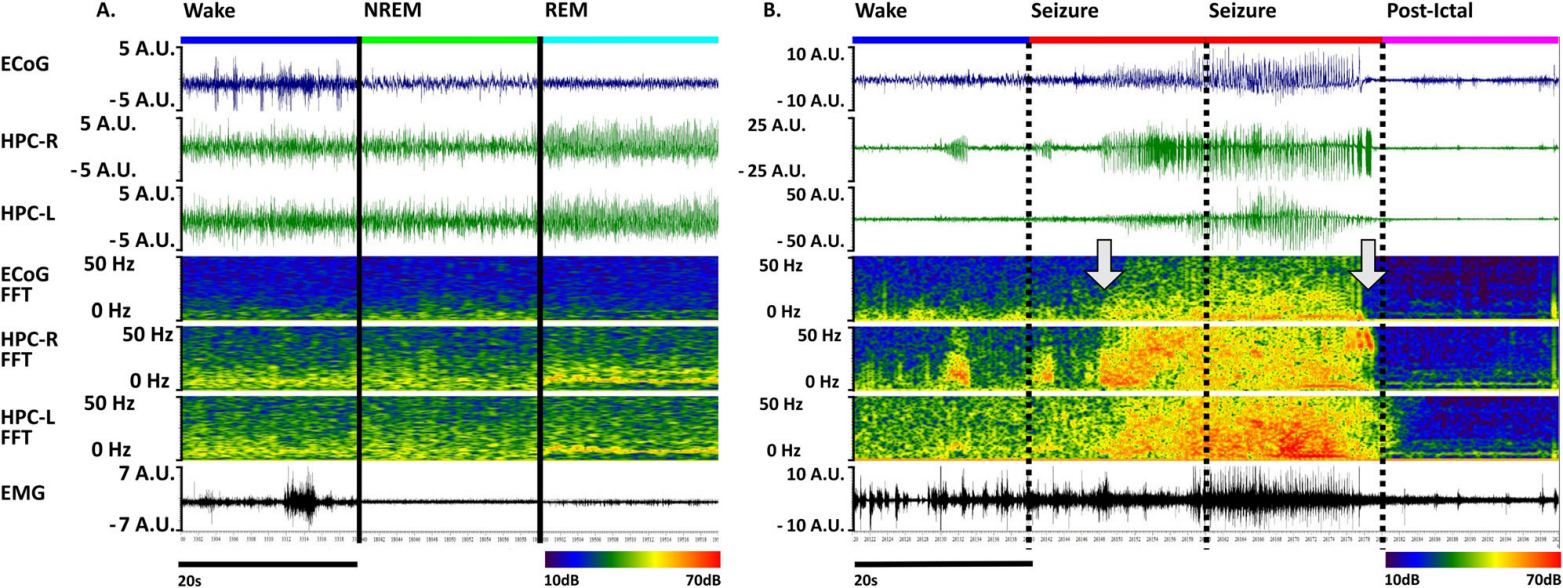
Scoring of sleep–wake states and seizures. ***A***, Sleep–wake categorization on three nonconsecutive 20 s epochs drawn from ECoG, left and right hippocampus (HPC-L and HPC-R), and EMG channels. *Y*-axes are presented in arbitrary units (a.u.) to reflect potential voltage range differences between mouse cohorts. The use of normalization in the preprocessing pipeline addresses concerns about using specified units on inputs. ***B***, In the same channels, a spontaneous seizure occurs from wake in the IAKA mouse model, divided into 20 s consecutive epochs, followed by postictal obtundation. A spike train begins in the left hippocampus during the epoch labeled “wake,” progresses into a full seizure before the midpoint of the second epoch (left arrow), and ends just before the third epoch ends (right arrow), resulting in a label of “seizure” for the second and third epochs. The fourth epoch shows the suppressed electrographic signal characteristic of a postictal state. The magnified image of this seizure can be found as Figure 2.

**Figure 2.**
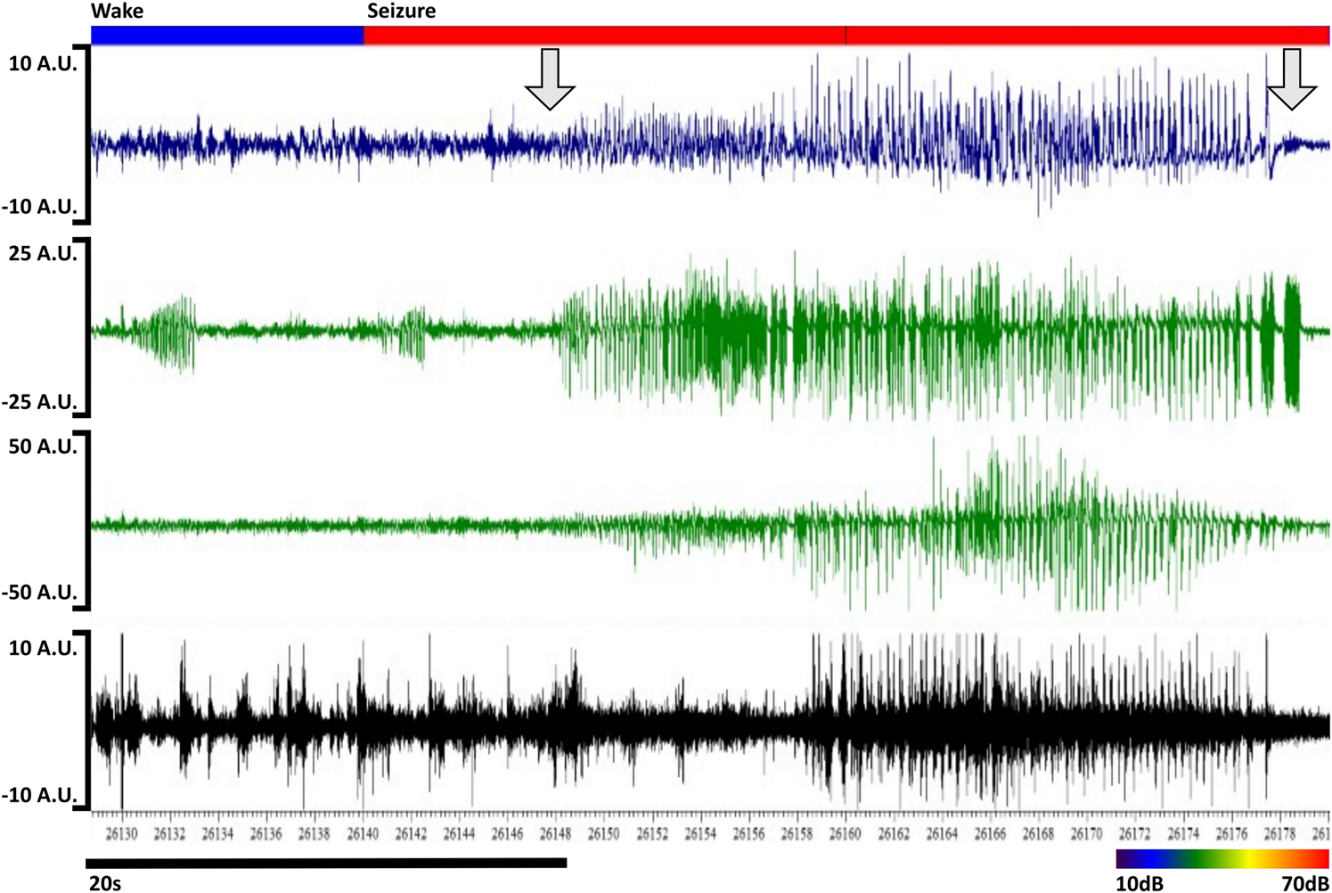
Example seizure from an IAKA mouse. The seizure from Figure 1*B* is shown magnified for detail and clarity.

Each 20 s epoch was assigned one of five possible labels [wake, rapid eye movement (REM), non-REM (NREM), seizure, or the postictal state; Figs. 1, 2] when half or more than half of the epoch consisted of that state except in the case of seizure epochs. Sleep–wake states were labeled by conventional criteria. Wakefulness is characterized by variable and predominantly theta through gamma EEG activity, often with phasic EMG activity. NREM is characterized by high delta power and loss of beta and gamma activity in the EEG, along with lower EMG activity than wakefulness. NREM sleep was not divided into substates as it is in humans, which is typical for rodent scoring (Rayan et al., 2024). REM is associated with lower EMG activity than non-REM, low delta EEG activity, and high theta activity, particularly in hippocampal electrodes, with the latter becoming less prominent in some epileptic mice.

Scoring was adjusted based on rules for mouse sleep adapted from American Academy of Sleep Medicine (AASM) and Rechtschaffen and Kales criteria (Rechtschaffen and Kales, 1968; Moser et al., 2009): REM can only follow NREM, and NREM can only be scored when there are two or more consecutive epochs.

Seizure scoring was performed via visual EEG scoring that required 5 s or more of rhythmic spikes that were evolving in morphology, frequency, or amplitude (Figs. 1, 2). Epochs that contained the bulk of the seizure, for short seizures, or included >5 s of seizures were scored as “seizure.” The rationale for this is that seizures are typically of high spectral power and dominate the epoch's normalized feature vector (see below). Seizures in rodents typically dominate the EEG signal, making sleep–wake scoring fraught, and are likely associated in many cases with impaired awareness. Thus, seizures were scored as a distinct state from sleep–wake states. The postictal state is characterized by initial behavioral obtundation and postictal electrographic suppression but can be scored without reference to video. This state is marked and remits suddenly, so visual scoring was used (see below for agreement between scorers). This state was included given that it is a state of behavioral obtundation and/or forebrain dysfunction that does not fit into typical sleep–wake states.

### Dataset composition

The dataset, including unscored data, contains 3,770 files (1,885 d) from experimental IAKA mice and 650 files (325 d) from either pretreatment baseline recordings or saline-injected controls. Of those, a total of 900 files (450 d) from experimental IAKA mice and 279 files (139.5 d) from either pretreatment baseline recordings or saline-injected controls had been manually scored (∼27% of the dataset). Data containing either uninterpretable recording errors [i.e., values that are “not a number” (NaNs) after computation] or text-encoded markers containing sleep scores that were not part of the target scoring states (such as markers for scoring on which experts disagreed) were not used to ensure error-free computation. Three files were excluded by these rules, leaving a final total of 1,176 files.

### Computational resources

Machine learning was implemented using the Python libraries TensorFlow (Google, version 2.10.0;  Abadi et al., 2015) and Keras (version 2.10.1; Chollet, 2015), with GPU training and inference using cuDNN version 8.1 and Cudatoolkit version 11.2.2 (NVIDIA et al., 2021). These versions were used to ensure forward Windows compatibility for future development of end-user tools. The class distribution contained several imbalanced classes, which were accounted for using the SciKitLearn (version 1.0.2; Pedregosa et al., 2011) compute_class_weight function (see below, Statistics and classification metrics). All file conversion, feature extraction, model training, and model inference were performed on a desktop PC (Intel i7-6950X at 3.00 GHz, 128 GB RAM, Nvidia RTX4090 24 GB).

### Preprocessing

**Figure 3.**
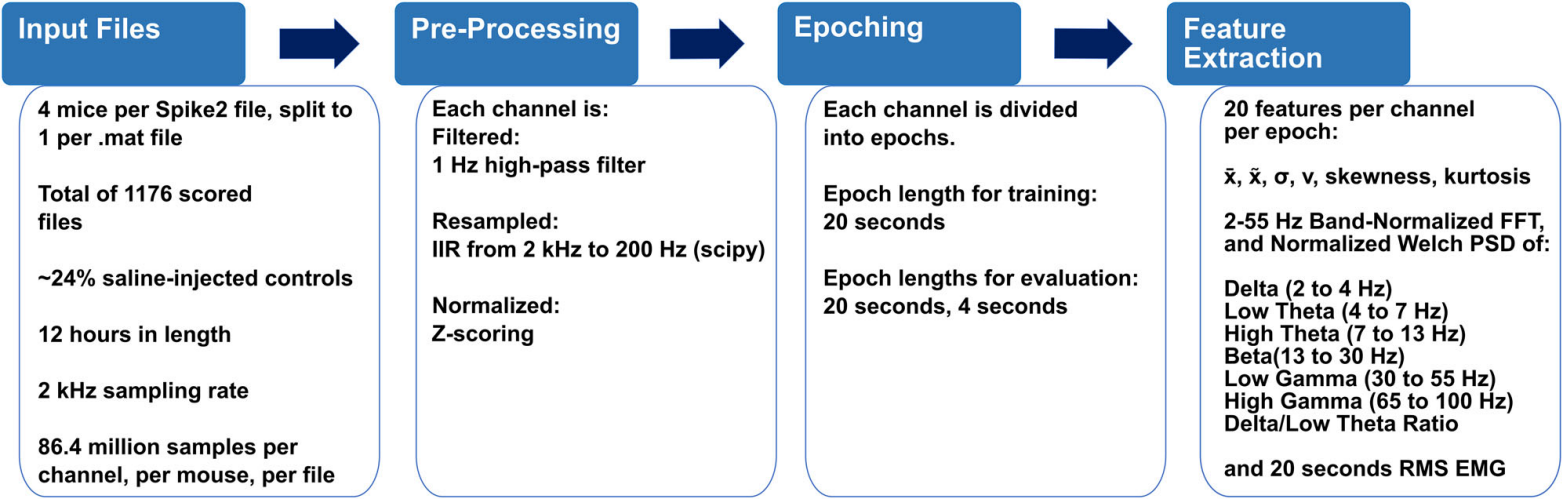
Data prepreprocessing pipeline in Python. Recorded data, as in Figure 1, is stored at a 2 kHz sampling rate as .smrx, an EEG-specific file format. As these files are large and not easily read into Python, they must first be exported with 20 s epochs to MATLAB .mat format via a script built in Spike2, the recording and analysis software. The .mat files are then downsampled by a factor of 10, including a zero-phase infinite impulse response anti-aliasing filter implemented in Scipy, then *z*-scored for normalization, parsed into a numpy array of shape (2,160, 4, 4,000) corresponding to (epoch count, channel count, samples) for 20 s epochs, or (10,800, 4, 800) for 4 s epochs. The decimated epoched array is then exported to .npy to further save disk space. Finally, feature extraction is performed on this decimated array on a per-epoch basis. Further information about how these features are used is available in Figure 4.

A custom script exported files for each mouse from Spike2 into MATLAB format. A Python Jupyter notebook was then used to perform the following preprocessing steps. Files were imported from the .mat format using HDF5Storage (version 0.1.18); then data were filtered on a per-channel, per-file basis using a first-order Butterworth high-pass filter at 1 Hz, using Scipy (version 1.7.3). Decimation was performed using Scipy's signal package to resample the data from 2 kHz to 200 Hz, including a second-order infinite impulse response anti-aliasing zero–phase filter for ECoG and an eighth-order version of the same filter for the HPC-L, HPC-R, and EMG channels. *Z*-scoring on a per-channel, per-file basis was then performed to normalize amplitudes. Numpy (version 1.21.6) was used to order the data into an array of 20 s epochs and save the data in the .npy format. Feature extraction was then performed via Numpy's real fast Fourier transform (FFT; Frigo, 1999) and the Scipy Signal Welch power spectral density (PSD) function (see below, Feature extraction; Fig. 3).

### Feature extraction

Six statistical features for each channel were extracted regarding the time-domain information of the signal within each epoch: mean, median, standard deviation, variance, skewness, and kurtosis. These statistical features were selected for their physiological relevance: median, standard deviation, and variance are commonly extracted features for EEG analysis (Stancin et al., 2021). Skewness and kurtosis were chosen as additional features due to their specific relevance and demonstrated effectiveness in EEG signal processing in epilepsy (Xiang et al., 2020).

Additionally, spectral features were calculated for each canonical frequency range of the EEG: delta (*δ*, 2–4 Hz), low theta (*θ*, 4–7 Hz), high *θ* (7–13 Hz), beta (*β*, 13–30 Hz), low gamma (*γ*, 30–55 Hz), and high *γ* (65–100 Hz). While line noise was low, given the recording configuration, we excluded 55–65 Hz to ensure this classifier did not have line noise-related problems if used in other settings. We computed the absolute magnitudes of the real portion of FFT and Welch PSD. Both FFT and PSD were used, as PSD is normalized to the width of the frequency range over which it is calculated. This provides a more accurate gauge of the relative power in bins of differing frequency widths, as we have in our paradigm. Each of these power estimates was normalized to the broadband FFT or PSD in the 2–55 Hz range to ensure that all spectral power measures were normalized relative to the baseline power of the epoch of interest. A ratio of delta power to low theta power was also calculated for both FFT magnitude and PSD, paralleling a primary feature used for manual scoring (often called “theta/delta ratio” or theta:delta). In total, 20 features were gathered for each of the four channels. Finally, 20 1 s RMS amplitude values are calculated from EMG for each epoch and concatenated with the other channel features for a total of 100 features per epoch.

**Figure 4.**
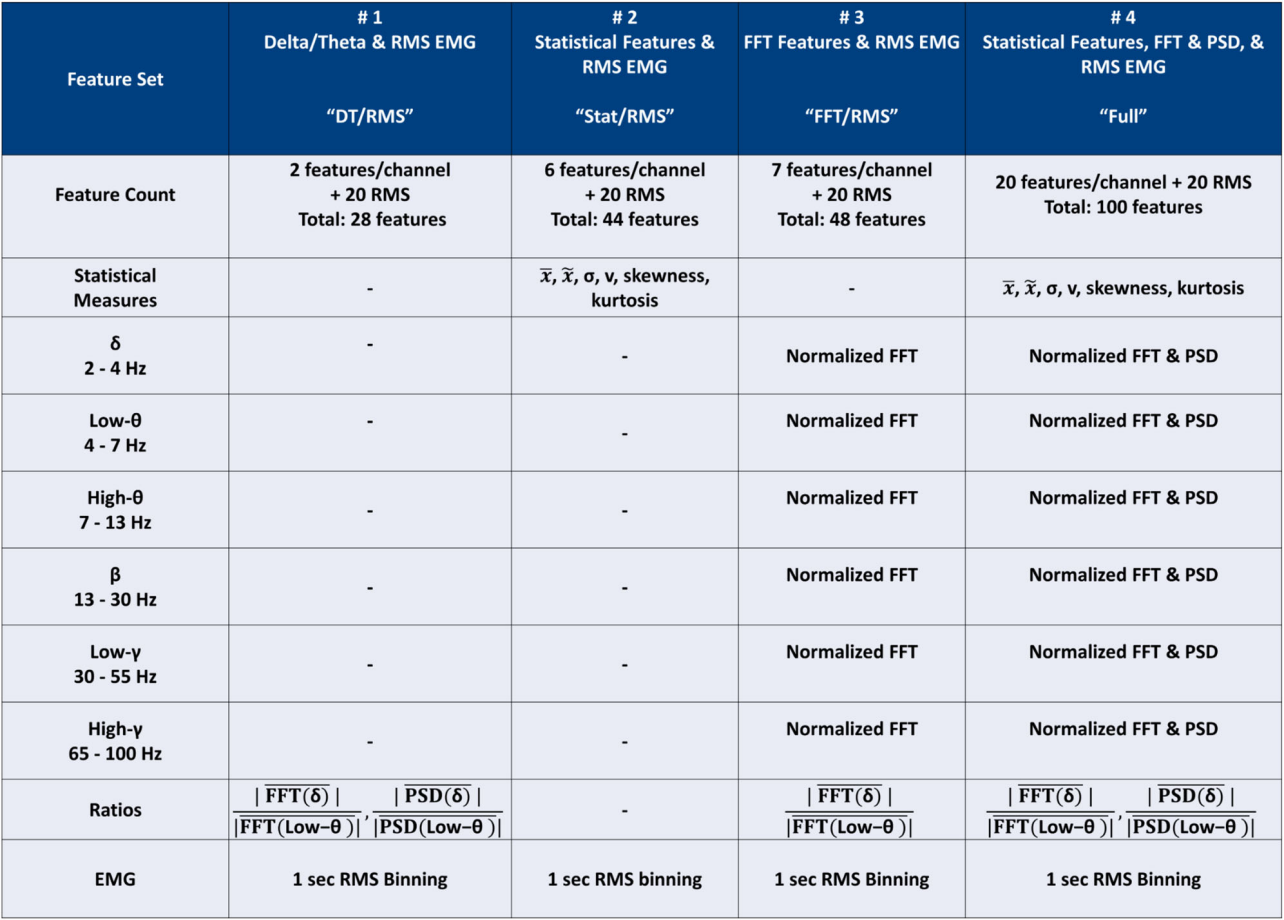
Feature set selection. Testing the feature space dependence of the various classification regimes was accomplished by selecting three groups of features to compare. The first (DT/RMS) consisted solely of the four channels’ delta/theta ratios in both FFT magnitude and PSD, as well as the full vector of RMS EMG amplitude. This most closely reproduces the features most important to a sleep–wake expert scorer when scoring manually. The second feature set (Stat/RMS) included seven statistical features per channel for each epoch, as well as the RMS EMG amplitude. The third feature set (FFT/RMS) included only the four channels’ Fourier magnitudes in the selected bins (normalized to broadband 2–55 Hz magnitude), the delta/theta ratio, and the RMS EMG. No epoch-level statistical features nor PSD were used for Feature Set 2. The final evaluated feature set contained all statistical features, FFT and PSD magnitudes (both normalized to their relative 2–55 Hz broadband magnitudes), delta/theta ratios for FFT and PSD, and RMS EMG components of the full 100-feature vector and is referred to in subsequent figures as the Full feature space.

The above features were then split into groups which were compared with validate our feature selection and provide some direct comparison to manual scoring where applicable. The first consisted solely of the four channels’ theta:delta in both FFT magnitude and PSD and the full vector of RMS EMG amplitude for the epoch. This closely reproduces the commonly used features for manual sleep–wake scoring. We refer to this first feature set as “Delta/Theta and RMS” (DT/RMS). As previous classifiers based primarily on these features have worked in nonepileptic animals, this was a key feature to include to demonstrate the need for a more advanced classifier for the analysis of kainic acid-treated animals. Feature Set 2 consisted solely of the six statistical features for each channel in the epoch and RMS EMG—a total of 44 features. Feature Set 3 included only the four channels’ Fourier magnitudes in the selected bins, the delta/theta ratio, and the RMS EMG. No epoch-level statistical features nor PSD were used for Feature Set 3, referred to as FFT/RMS. The final evaluated feature set contained all statistical features, FFT and PSD magnitudes, delta/theta ratios, and RMS EMG components of the full 100-feature vector and is referred to in subsequent text and figures as the Full feature set. See Figure 4 for a visual summary of tested feature sets and frequency bins of interest.

Extracted features for each epoch were input to ScikitLearn and Keras models in array format, with each feature vector in the input *x* array corresponding to an epoch label at the same index in the *y* array. As epochs were exclusively scored as one of five states, *y* label arrays were encoded as one-hot labels with five indices. These indices correspond to (wake, NREM, REM, seizure, postictal). For example, an epoch scored as wake would be represented as [1, 0, 0, 0, 0].

### Training, validation, and test dataset creation

The data were fully separated into training/validation/testing datasets on a per-subject basis to avoid any in-sample training. Data used for training contained animals of all types, with ∼97.3% being VGAT-Cre IAKA animals (*n* = 34) and ∼2.7% being VGAT-Cre saline control animals (*n* = 1). The overrepresentation of VGAT-Cre IAKA animals was intentional, as this phenotype has marked intersubject variability in the power of frequency bands typically used to detect sleep. The validation dataset used to assess loss functions during training contained solely VGAT-Cre saline control animals (*n* = 3). This was also an intentional choice, as the main function of this validation dataset was to ensure sleep scoring generalization between both the IAKA and saline-treated animals. The holdout testing dataset consisted of ∼35% VGAT-Cre IAKA (*n* = 7), 50% wild-type IAKA (*n* = 10), and ∼15% wild-type saline controls (*n* = 3). In building this additional testing dataset from these recent cohorts, we can ensure that the classifier generalizes fully to a different genotype and to the introduction of novel cohorts of animals. Of the 1,176 scored 12 h recordings, the final file split was ∼60.12% (*n* = 707 files) for training, 10.97% (*n* = 129) for validation, and 28.91% (*n* = 340) for testing the final versions of the evaluated models. This data split was effective for training with manually scored data while reliably producing an accurate classification on completely out-of-sample validation animals.

### Model architectures

We selected several approaches to machine learning that were based in either what we took to be the implicit processes involved in the human scoring, mirrored nonmachine learning approaches, or adopted architectures that had previously been found to be effective. We started by examining the performance of a highly effective classifier of sleep–wake in nonepileptic mice, “AccuSleep”, that is open-source and thus configurable for our dataset. Next, a SVM approach was used as a benchmark, given that a discriminant function is used or implicit in manual and spreadsheet approaches’ reliance on theta:delta and RMS EMG We hypothesized this would not perform well for epileptic mice, despite reasonable classification for controls given disrupted theta:delta due to background slowing and disrupted theta in epileptic mice. The other approaches were based on multilayer neural network models that had previously been shown to be effective for automated sleep scoring in humans or rodents. To compare methods, we trained several varieties of these models on our three sets of extracted features and then compared the performance of these models with validation and test datasets to determine the best model for our application.

#### AccuSleep

We first wanted to determine how an effective sleep–wake classifier would perform with epileptic mice. We used the open-source AccuSleep framework (Barger et al., 2019), given that it could be directly compared with the structure of our data and retrained as necessary. Briefly, AccuSleep is a multilayer convolutional neural network that classifies images of the spectrogram of log-normalized spectra for each epoch, in addition to normalized RMS EMG. Labels of wake, NREM, REM, or unknown are assigned with customizable epoch lengths. AccuSleep can be downloaded pretrained with various epoch lengths and used with custom epoch lengths by pooling its native shorter epochs. AccuSleep does not feature a pretrained model with a 20 s epoch length, so we evaluated AccuSleep's performance with our ground-truth labels in two ways. First, we tested the pretrained model with a 10 s epoch length against the 340 manually scored files from the holdout testing dataset, splitting each 20 s epoch into 10 s labels, to directly compare its performance with the performance of our classifier variants. We then retrained AccuSleep with 20 s epochs and manually scored labels from all files from our training dataset (705 files) and tested against our entire holdout testing dataset.

As during retraining AccuSleep ignores any epochs labeled as “Unknown,” this fourth class will only be shown in confusion matrices to illustrate how AccuSleep scores our seizure/postictal epochs, and no inferences will be made about any ability of AccuSleep to evaluate seizure.

#### SVM

The baseline architecture for comparison to human scoring was a SVM architecture (Vapnik, 1997) with a linear discrimination function. SVMs, simply, take a set of (*y*, *x*) points (*y* is a class label, and *x* is a feature vector) and map them to points (*y*, *z*) in a higher-dimensional feature space *Z*, where the label *y* corresponds to a derived set of features *z* in the high-dimensional space. The classification problem is then solved by the SVM, by determination of a hyperplane which maximally separates the sets of points within this higher-dimensional feature space. The SVM used herein relies on a hinge loss function for multiclass classification (Crammer and Singer, 2001). The SVM, trained with an iteration count of 1,000 using SciKitLearn's linear_model.SGDClassifier function with hinge loss and L2 regularization, was trained and validated against all four feature sets, using class weights to correct for class imbalance.

#### Multilayer architectures

We selected four multilayer architectures for classification that were either commonly used architectures or had been shown as effective for sleep–wake classification. (1) Dense (fully connected) layers: dense layers are the simplest starting point for a neural network, being fully connected layers that return the dot product of their received inputs and the weights learned by the layer's kernel. Dense layers used herein operated on a linear activation function. The use of this architecture is a baseline, as this is a widely used and relatively straightforward architecture, and its success or failure in classification is used to evaluate the necessity for more complex architectures. Dense layer architectures were implemented with a dense layer (of variable size) to perform the tensor operations on the input sequences, a flatten layer used to compress the sequences from three dimensions to two dimensions, and a five-way softmax output layer used in the grid search for hyperparameter tuning. Long short-term memory (LSTM): LSTMs are a type of recurrent neural network where three gates (input, output, and forget), along with input from previous time steps, are leveraged to control which learned weights are remembered from past predictions and used to predict the current time step (Hochreiter and Schmidhuber, 1997). LSTM implementation: LSTM architectures were implemented with an LSTM layer (of variable size), a 40% dropout layer for regularization, a flatten layer, and a five-way softmax output layer. BiLSTM: BiLSTMs are a variant of LSTM layers that perform their forget-gate operations on time steps in both the forward and backward directions, as opposed to the solely backward-looking operation of LSTMs. This provides much more utility in the context of predicting labels in cases where the signal characteristics of time points later in the signal are known, as is the case in offline vigilance and ictal state scoring (Graves and Schmidhuber, 2005). BiLSTM architectures were implemented with a BiLSTM layer (of variable size), a 40% dropout layer, a flatten layer, and a five-way softmax output layer. We also implemented a stacked variant of the BiLSTM (Stacked-BiLSTM), with each of four BiLSTM layers halving in size as they progress. The first layer in the chain is of variable size. The final BiLSTM layer was then used as input to a 40% dropout layer, a Flatten layer, and a five-way softmax output layer.

### Grid search paradigm for multilayer architectures

Training was executed using each possible combination of various manually specified parameters to tune the model's inputs and features. The first feature tested in our grid search of models was the three feature vectors. The second feature tested in this grid search was the number of units in the variable-size base layer of each architecture at variants of 50, 100, and 200 units. For testing purposes, the same layer size input was used in LSTM as in BiLSTM, resulting in the first layer of the BiLSTM architectures consisting of (two times the layer size) input units, where the layer size is denoted in figures and tables. Our third feature evaluated was input sequence length, where the epoch of interest was input in sequence with several preceding and following epochs. The assessed variants of input sequence length were 1, 3, 5, and 7. For example, at sequence length 1, epoch *x_T_* is paired with epoch state label *y_T_*. At sequence length 7, epoch *x_T_* is presented to the classifier as the vector {*x_T_*_ − 3_, *x_T_*_ − 2_, *x_T_*_ − 1_, *x_T_*, *x_T_* _+_ _1_, *x_T_* _+_ _2_, *x_T_* _+_ _3_}, with the state label *y_T_*. Our use of an input sequence of 1 allows for the evaluation of the architectures’ scoring metrics without the benefit of temporal context. The use of input sequences of varying lengths is standard with the use of LSTM classification and is the equivalent of a sliding-window feature extraction approach.

**Figure 5.**
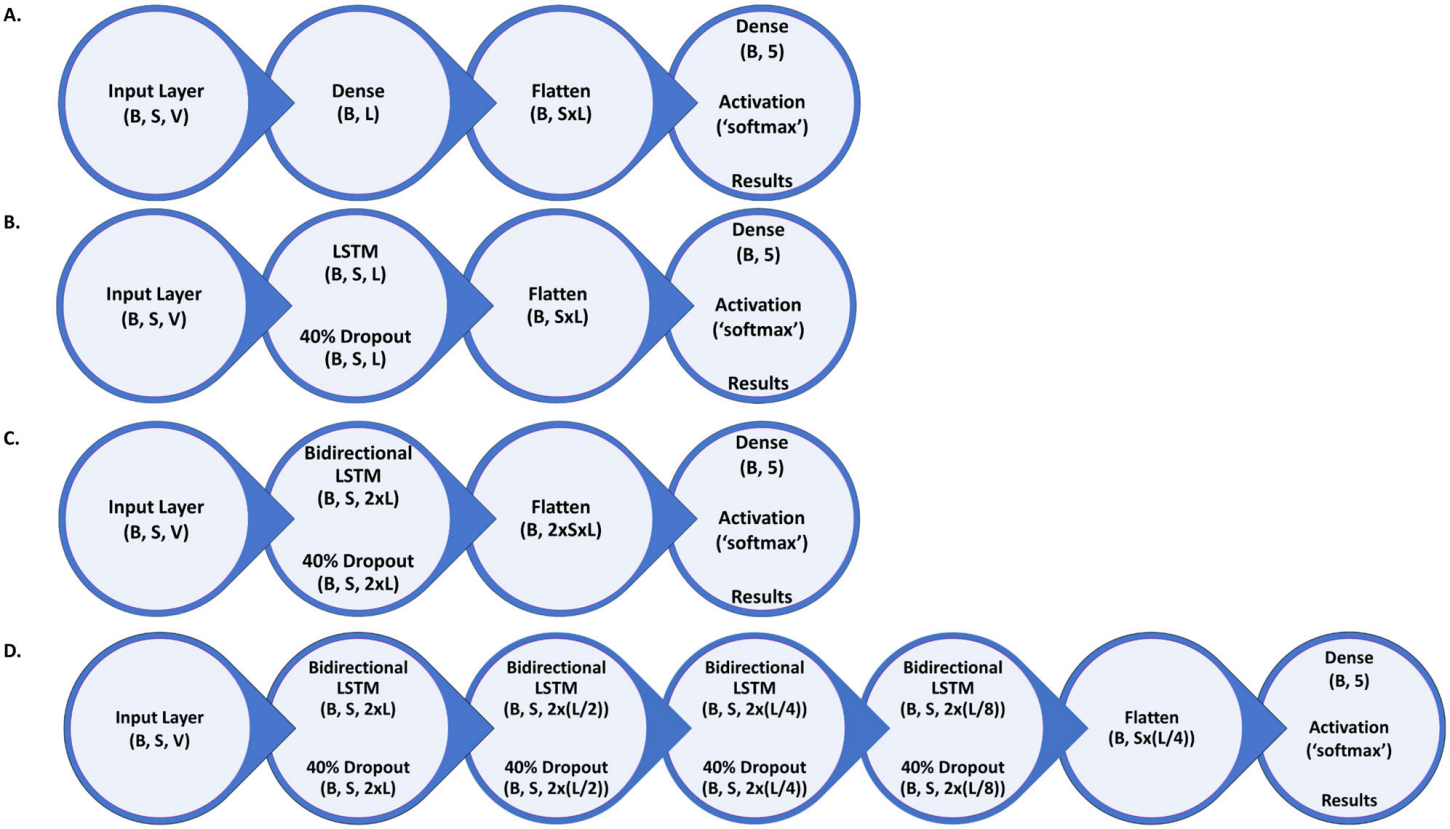
Layer architecture search space. Graphical representation of the layer architectures tested and the parameters applied. Variables tested in the grid search are represented here with single letters. B represents the batch size of 2,160, that is, the length in 20 s epochs of one full 12 h electrographic recording. S represents the variable sequence length of epochs in the input vector. V represents the variable length of the input vector itself. L represents the variable layer sizes of 50, 100, or 200.

The initial grid search consisted of a combinatorial search of four feature vectors, four machine learning models, three-layer sizes, and four input sequence lengths. With the addition of the four feature vectors tested for the SVM evaluation, the total number of models evaluated in the initial grid search was 196. To reduce computational time, 20 training epochs using the full training data were performed for the first round of evaluation before classification matrices, and reports were saved. The early stopping criterion for all training was decided to be when there was a lack of change of 0.001 in loss function in five training epochs. Examples of layer architectures, with all assessed variables represented in the architecture diagrams, are presented in Figure 5 and the accompanying legend.

### Statistics and classification metrics

Both two-way ANOVA and Welch’s *t* test were employed using GraphPad Prism 10.5.0 on running on Windows 10 to perform between-genotype comparisons in seizure rates and total spontaneous seizure burden to test the similarity of IAKA-induced seizure phenotypes.

A modified version of the output from the SciKitLearn compute_class_weight method was used to provide class weight inputs for the imbalanced classes to Keras. The weights generated by this function are used as input to the class-weight parameter of Keras and are used to assign a relative weight to each class with respect to their impact on the loss function of the machine learning model. The compute_class_weight function was used with the parameter “balanced,” via the following equation:

$$\mathrm{SK}\_\mathrm{Weigh}t_{C}=\frac{N}{T\;*\;N_{C}}\;$$

In Equation 1, *N* denotes the number of samples, *N_C_* denotes the number of samples of a given class, *T* denotes the total number of classes, and SK_Weight*_C_* is the value returned for that class by the compute_class_weight method. This class weighting function, however, gives a very large range of values for classes where the counts differ by orders of magnitude, causing Keras to operate inefficiently. To overcome this limitation, each number in the resulting class weight array was then modified via the following equation to smooth the values in the class weighting array while maintaining their relative scales:

$$\mathrm{Weigh}t_{C}=\ln(\mathrm{SK}\_\mathrm{Weigh}t_{C})\;-\;\ln(\mathrm{SK}\_\mathrm{Weigh}t_{\mathrm{wake}}\;)\;+\;1$$

Equation 2 sets the weight of wake to 1 and scales the rest of the weights relative to ln(SK_Weight_wake_).

Using Keras’s built-in metrics methods, the following classification metrics were calculated for each epoch of training: true/false positives, true/false negatives, categorical accuracy, precision, recall, area under the precision-recall curve (AUCPR), and categorical cross-entropy loss:

$$\mathrm{Precision}=\frac{\mathrm{TP}}{\mathrm{TP}+\mathrm{FP}}\;\;$$

$$\mathrm{Recall}=\frac{\mathrm{TP}}{\mathrm{TP}+\mathrm{FN}}$$

The AUCPR is approximated as the Riemann sum of a plot of precision versus recall values at 200 different thresholds, all of which are calculated for each one-hot encoded state for a given epoch. Categorical cross-entropy loss is a loss function used to optimize and evaluate the classifier. This loss function is calculated by the cross-entropy function (Liu et al., 2020):

$$\mathrm{loss}=\;-\overset{N}{\underset{n=1}{\sum }}\;{\hat{y}}_{i,C1}\log(y_{i,C1})+{\hat{y}}_{i,C2}\log(y_{i,C2})+{\hat{y}}_{i,C3}\log(y_{i,C3})+{\hat{y}}_{i,C4}\log(y_{i,C4})+{\hat{y}}_{i,C5}\log(y_{i,C5})$$

In Equation 5, *y_i,C_*_1_ to *y_i,C_*_5_ represent each of the labels in the one-hot encoding for a given sample, and *ŷ_i,C_*_1_ to *ŷ_i,C_*_5_ represent the five outputs from the five-way softmax output layer. In all of the machine learning models used, we optimize the loss function using the *N*adam optimizer (Dozat, 2016).

SciKitLearn's built-in metrics methods were also used to produce true/false positives, true/false negatives, categorical accuracy, precision, and recall for the purposes of output to Excel format. SciKitLearn also allowed for the calculation of the F1 score for each class given by the following equation:

$${F1}_{\mathrm{Micro},C}=2*\frac{\mathrm{Precisio}n_{C}\;*\;\mathrm{Recal}l_{C}}{\mathrm{Precisio}n_{C}\;+\;\mathrm{Recal}l_{C}}$$

In Equation 6, F1_Micro.*C*_, Precision_C_, and Recall_C_ denote the score calculated for that class. These scores were calculated via the SciKitLearn classification_report function using the true and predicted labels for each epoch, determined by the class with the highest-scoring prediction probability. F1_Micro_ scores will be used for cross-model analyses except in cases where precision or recall is substantially different from, and thus not properly summarized by, F1_Micro_.

The macro and weighted multiclass F1 scores were also calculated by the SciKitLearn classification_report function according to the following equations, where *N*_Classes_ is the total number of classes identified and *N* is the total number of samples:

$$F1_{\mathrm{Macro}}=\;\frac{F1_{\mathrm{Micro},C1}+F1_{\mathrm{Micro},C2}+F1_{\mathrm{Micro},C3}+F1_{\mathrm{Micro},C4}+F1_{\mathrm{Micro},C5}}{N_{\mathrm{Classes}}}\;\;$$

$$F1_{\mathrm{Weighted}}=\frac{\;(N_{C1}\;*\;F1_{\mathrm{Micro},C1})+(N_{C2}\;*\;F1_{\mathrm{Micro},C2})+(N_{C3}\;*\;F1_{\mathrm{Micro},C3})+(N_{C4}\;*\;F1_{Micro,C4})+(N_{C5}\;*\;F1_{\mathrm{Micro},C5})}{N}$$

Confusion matrices were created using SciKitLearn’s confusion_matrix function.

Mean false alarm rate (FAR) per hour for seizure detection was calculated for all files with manually scored seizures via Equation 9. Each file's expert-annotated seizure counts (Seizures_Expert,*i*_; separate runs of contiguous epochs of seizure) were subtracted from that file's classifier-detected seizures (Seizures_Classifier,*i*_) to calculate false alarms (FA*_i_*). This quantity was then divided by the recording length in hours of that file (*T_i_*) to give a FAR per hour for each file. Average FAR $\left(\bar{\mathrm{FAR}}\right)$ was then calculated by averaging this quantity across the evaluated datasets:

$$\bar{\mathrm{FAR}}=\frac{1}{N}\overset{N}{\underset{i=1}{\sum }}\;\mathrm{FAR}=\frac{1}{N}\overset{N}{\underset{i=1}{\sum }}\frac{FA_{i}}{T_{i}}=\;\frac{1}{N}\overset{N}{\underset{i=1}{\sum }}\frac{{\mathrm{Seizures}}_{\mathrm{Classifier},i}-{\mathrm{Seizures}}_{\mathrm{Expert},i}}{T_{i}}$$

Cohen's *κ* (Cohen, 1960) was employed to assess agreement between the manual 20 s scoring and the final classifier results, using the SciKitLearn cohen_kappa_score function. This function operates according the following equation:

$$\kappa \;=\frac{(\;p_{o}\;-\;p_{e})\;}{\;(1\;-\;p_{e})}$$

In Cohen's *κ*, *p_o_* indicates the observed probability of assignment of a label, and *p_e_* indicates the expected probability of assignment of a label. The use of this statistic is a conventional metric for assessing sleep scoring accuracies and has been included to provide a point of comparison for other sleep-classification researchers. It was not used for model evaluation during training, validation, or testing.

### Classification performance of trained classifier with shorter epochs

After optimal model selection based on the grid search, we sought to examine generalization of this classifier to shorter epochs. We selected 4 s epochs, as this epoch length is a common lower bound of rodent sleep scoring which allows for feasible manual scoring and as well as calculating spectral estimates for low frequency EEG activity. Twenty-second epochs are often used when raw amounts of sleep–wake are studied; 4 s epochs are more appropriate when examining sleep fragmentation or narcolepsy models with brief cataplexy. The change of epoch length required a modification to the signal preprocessing to adapt RMS EMG to the feature vector. The RMS EMG was calculated as before, in 1 s steps. This vector of four 1 s bins, {RMS_1_, RMS_2_, RMS_3_, RMS_4_}, was then distributed to the 20-feature RMS EMG vector with five repeats per RMS value. This repetition allows the 1 s signal to fit the existing 20 s feature vector.

## Results

### Dataset composition

A total of 21 of 79 animals were excluded from this analysis. The first exclusion criterion was death prior to IAKA administration (*n* = 1, VGAT-Cre). The second was epilepsy-related death on the day of IAKA administration (*n* = 9; seven VGAT-Cre, two wild types). The third was death in the short term (4 d) after recording (*n* = 3, VGAT-Cre). The fourth was technical failure (*n* = 5; four VGAT-Cre, one wild type) before the study end point 3 weeks after kindling. The fifth was membership in the first experimental cannula cohort (*n* = 4, VGAT-Cre). Files from VGAT-Cre mice that died within 4 d of IAKA were not manually sleep scored as the limited number of files in these partial recordings. One VGAT-Cre mouse and one wild-type mouse which died within 5 d of IAKA administration were included based on membership in our most recent cohorts targeted for the testing dataset. These files only represented 3 of 340 files in the testing dataset.

Of the five animals that had technical failures which were not processed for this experiment, the specific subgroups include known bad signal in one or more channels (*n* = 1, wild type), broken injection cannula preventing clear assignment to an experimental group (*n* = 2, VGAT-Cre), and sudden complete loss of signal in one or more electrodes (*n* = 2, VGAT-Cre). A full accounting of exclusion reasons per animal, recording lengths and details, and files used for each dataset grouping can be found as Extended Data 1, under the file name “Animal Statistics and Group Information.xlsx.” There were 58 animals remaining after all exclusion criteria, with 8,484 h of recording from 35 VGAT-Cre animals included in the training dataset, 1,548 h from 3 VGAT-Cre animals in the validation dataset, and 4,080 h from 20 animals (*n* = 13, wild type; *n* = 7, VGAT-Cre) in the testing dataset.

### Seizure rates by genotype

When analyzing spontaneous seizures occurring outside of status epilepticus (Days 2–21 after IAKA administration), daily seizure count for VGAT-Cre animals (mean of 1.48 seizures per day) and wild-type animals (mean of 0.94 seizures per day) are not significantly different when analyzed via two-way ANOVA for genotype (df = 1; *F* = 0.6052; *p* > 0.05), time (df = 19; *F* = 1.026; *p* > 0.05), or time × genotype interaction (df = 19; *F* = 0.3806; *p* > 0.05), with only the subject-level effects (df = 29; *F* = 6.111; *p* < 0.001) accounting for the variability in daily seizure counts. A Welch's *t* test for genotypic differences in total seizure count after IAKA was nonsignificant (*p* > 0.05). These results support a similar seizure phenotype after IAKA between our VGAT-Cre and wild-type animals.

### Performance of existing sleep–wake classifier (AccuSleep) in epileptic mice

**Figure 6.**
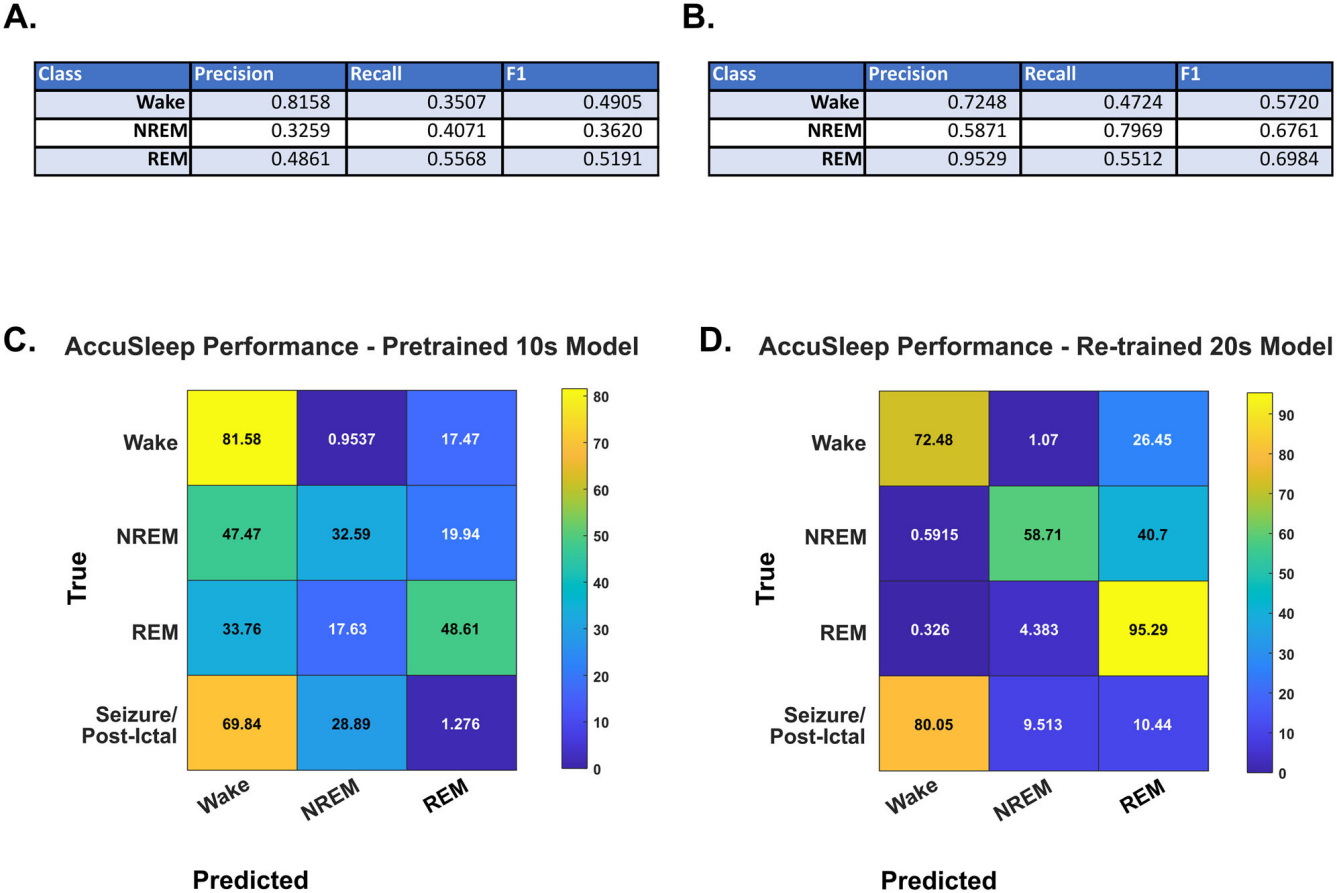
AccuSleep performance on epileptic mouse data. ***A***, ***B***, Precision, recall, and F1 values for each state for the published 10 s epoch (***A***) and retrained 20 s epoch (***B***) AccuSleep classifiers. This classifier does not have the ability to train or score seizure states, so the fourth class for prediction was read as “Unknown,” though these epochs were coded separately from the sleep states for the ground truth. ***C***, The confusion matrix of a pretrained 10 s epoch AccuSleep classifier evaluated on our holdout testing dataset described in Materials and Methods. It is evident that, while the classifier can precisely classify wake states, it does not have proper recall and is prone to false-positive wake states for both NREM and REM classes. ***D***, The confusion matrix of a customized version of AccuSleep trained on 20 s epochs of our entire training dataset and evaluated on the entire testing dataset as described in Materials and Methods. This version of the classifier can identify wake states as well as REM states but is prone to false-positive REM states for NREM epochs. This classifier also identifies 9.51% of seizures and postictal states as NREM and 10.44% as REM.

We hypothesized that existing sleep–wake classifiers would underperform with epileptic mice given interictal EEG abnormalities including with epileptiform discharges and alterations in the EEG background. We evaluated a highly effective classifier that could be modified to work with our data and retrained with data from epileptic mice. To evaluate AccuSleep generously, we employed both the published, pretrained 10 s epoch length classifier as well as a version retrained on our training data (see Materials and Methods) to classify 20 s epochs. The available classes for AccuSleep's output were wake, NREM, REM, and unknown. Ground-truth data for our seizure and postictal states were recoded as Unknown for retraining purposes. Precision scores on the testing dataset for the pretrained AccuSleep were 0.816 for wake; 0.326 for NREM; and 0.486 for REM. Recall scores for the pretrained AccuSleep were 0.351 for wake; 0.407 for NREM; and 0.557 for REM. F1_Micro_ scores for the pretrained AccuSleep were 0.491 for wake; 0.362 for NREM; and 0.519 for REM. While positive classifications (precision) were high for wake, identification of all epochs (recall) was low, and the overall performance was not acceptable for sleep–wake scoring (Fig. 6*A*,*C*).

Using our full training dataset and the provided training scripts from AccuSleep, we obtained noticeably better average performance. Precision scores for the newly trained AccuSleep on the testing dataset were 0.725 for wake, 0.587 for NREM, and 0.952 for REM. Recall scores for the newly trained AccuSleep were 0.472 for wake, 0.797 for NREM, and 0.551 for REM. F1_Micro_ scores for the newly trained AccuSleep were 0.572 for wake, 0.676 for NREM, and 0.698 for REM (Fig. 6*B*,*D*). Despite the improvement in overall precision with retraining, classification of all instances of each state (recall) was still too low for our purposes and did not reach our benchmark.

**Figure 7.**
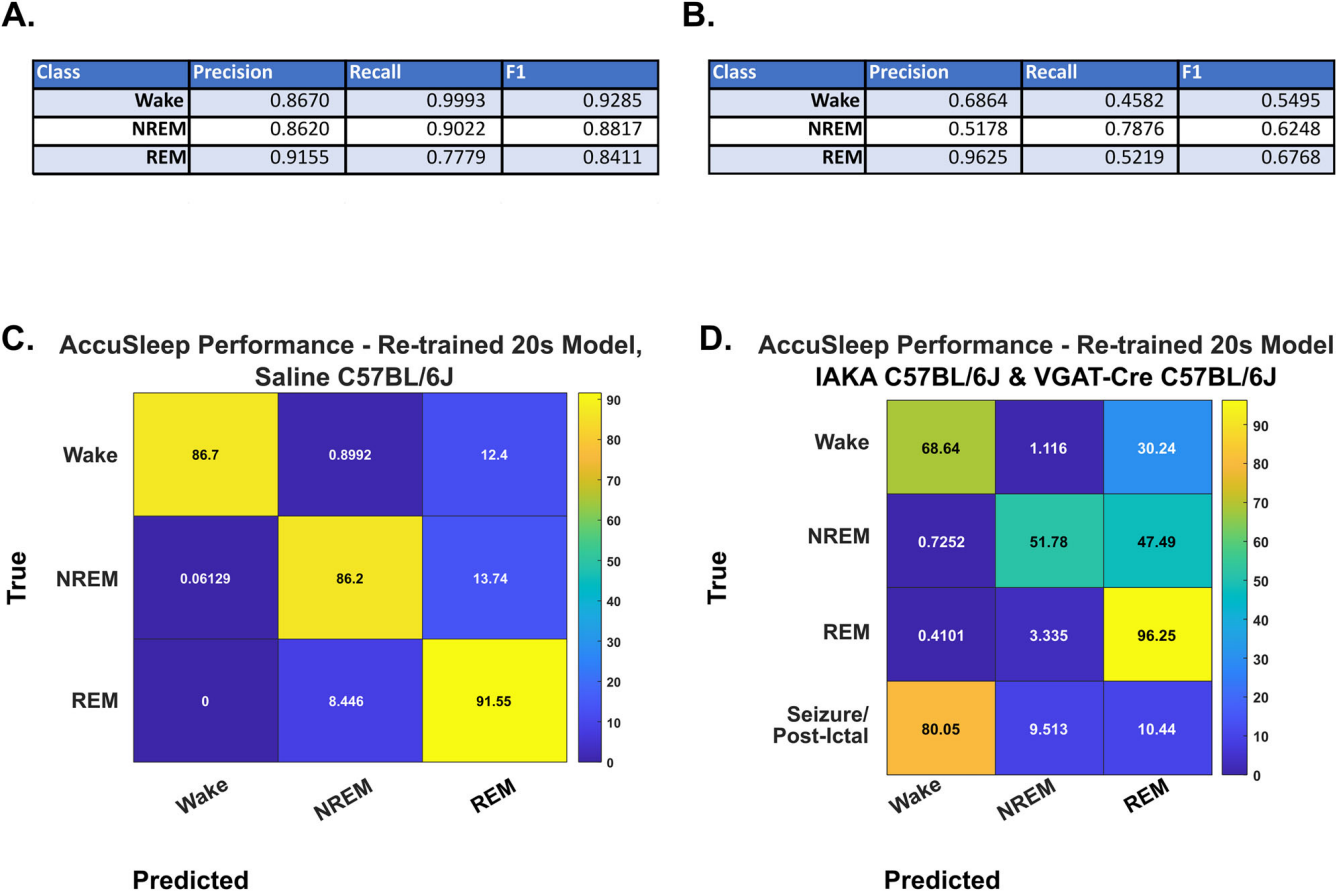
Retrained AccuSleep performance by condition. ***A***, ***B***, Precision, recall, and F1 values for each state for the 20 s epoch version of AccuSleep retrained on our training dataset for (***A***) saline-group wild–type animals from the testing dataset (*n* = 3) and (***B***) IAKA-group wild–type (*n* = 10) and VGAT-Cre (*n* = 7) animals. This classifier does not have the ability to train or score seizure states, so these were given a separate label, coded as “Unknown” in AccuSleep, for the ground truth. ***C***, The confusion matrix of the results from the retrained 20 s epoch AccuSleep classifier evaluated on 48 recording files featuring all sleep–wake states from the saline-group wild–type animals from the testing dataset described in Materials and Methods. This test demonstrates that our retraining methodology and testing of AccuSleep are valid for nonepileptic animals. ***D***, The confusion matrix of the results from the retrained 20 s epoch AccuSleep classifier evaluated on 292 recording files featuring all sleep–wake states, seizure, and the postictal state from the IAKA-group animals from the testing dataset. This version of the classifier can precisely identify REM states but misidentifies wake as REM 30% of the time and NREM as REM 47% of the time and, as in the previous AccuSleep test, cannot accept a class to identify our ictal and postictal states.

The division of our testing dataset into saline and IAKA groups yielded confirmation that our retrained model classifies saline animals much more accurately than IAKA animals, validating our training methodology while demonstrating the lack of classification in sleep states from the IAKA group. Precision, recall, and F1_Micro_ scores for all classes were lower for all sleep–wake states in the IAKA group than the saline group save for REM. The precision values were 0.867 for wake, 0.862 for NREM, and 0.916 for REM in the saline animals; for the IAKA animals, these values were 0.686 for wake, 0.518 for NREM, and 0.963 for REM. Confusion matrices and values for all metrics are presented in Figure 7.

For both tested versions of AccuSleep, as the design limited us to training on the three sleep classes, we can only evaluate the scoring of seizure/postictal epochs subjectively. What we can take from this portion of the experiment is that the pretrained model classified 69.84% of seizure epochs as wake, 28.89% as NREM, and 1.27% as REM. As the seizure is a state with pronounced EEG and EMG activation, this classification of seizure as wake in a model without seizure as a specific class would be expected. Likewise the model retrained on our training data classified 80.05% of seizure as wake, 9.51% as NREM, and 10.44% as REM. This performance of AccuSleep on our data corresponds with our suppositions about how the epilepsy associated changes in spectral character would cause existing sleep–wake classifiers to underperform on such animals. Given that this otherwise effective classifier underperformed for epileptic mice, we then compared some reasonable alternative approaches.

### SVM

**Figure 8.**
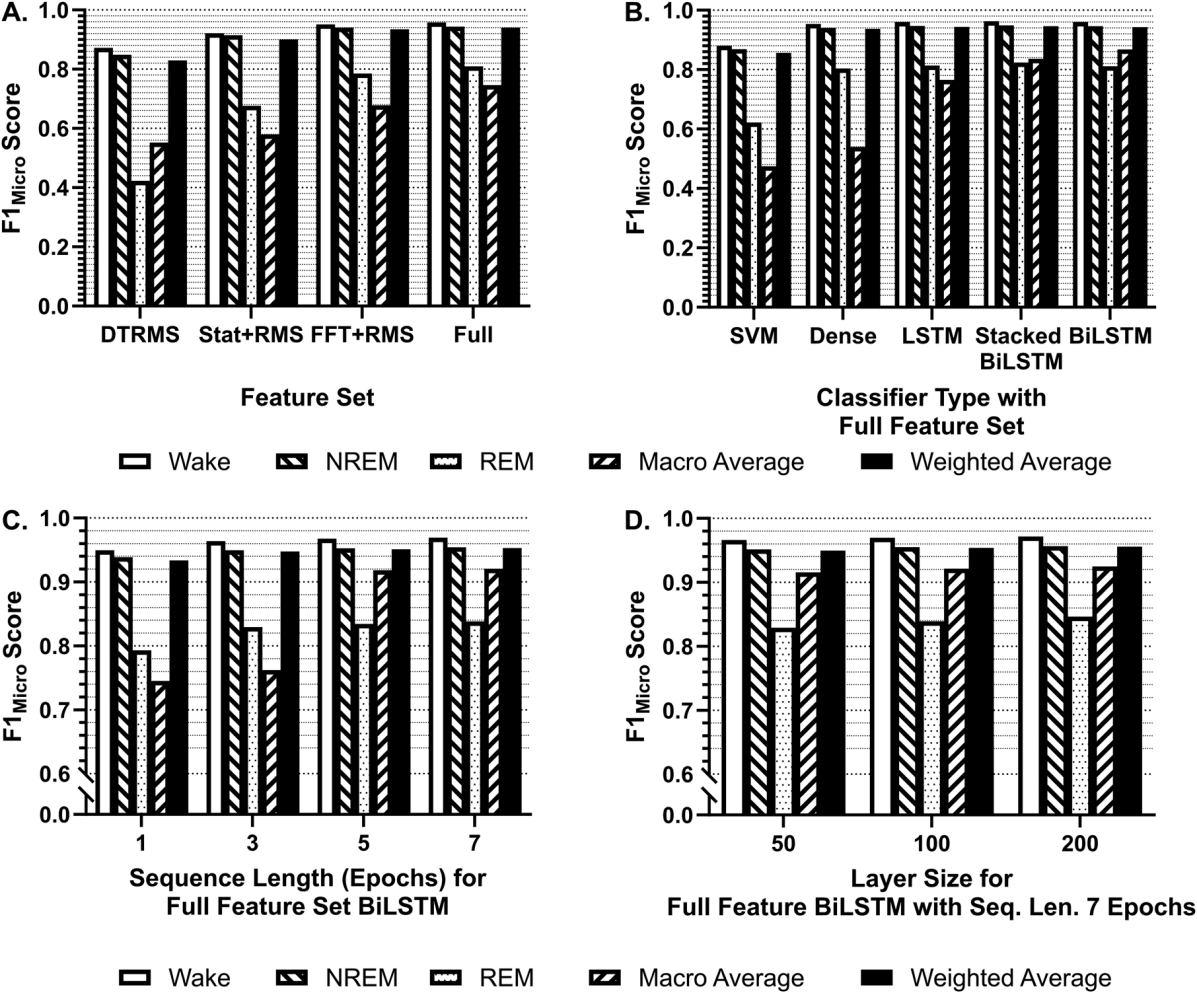
F1 metrics at 20 epochs of training. F1_Micro_ scores for the individual states contained in the validation dataset (wake, NREM, REM) as well as F1_Macro_ and F1_Weighted_ were assessed at each nested level of the grid search to determine the best-performing model. Each feature set, architecture, sequence length, and base layer unit count were exhaustively validated against one another. ***A***, For each feature set tested, each of the F1 metrics is averaged over all architectures, sequence lengths, and layer sizes. At this level of analysis, the greatest performance across all metrics was the Full feature set. ***B***, For each architecture tested using the Full feature set, each of the F1 metrics is averaged over sequence length and layer size. At this level of analysis, the greatest-performing classifiers were of the BiLSTM architecture. LSTM and Stacked-BiLSTM were comparable in performance. ***C***, For each sequence length tested as input to a BiLSTM architecture using the Full feature set, each of the F1 metrics is averaged over all layer sizes. At this level of analysis, the greatest-performing classifiers were trained with a sequence length of 7. ***D***, For each base layer unit count tested using seven-sequence-length inputs to a BiLSTM architecture using the Full feature set, each of the F1 metrics is averaged over all layer sizes. At this final level of analysis, the best-performing classifier on the test dataset in the 192-model grid search is the 200 base unit count, seven-sequence-length inputs, and BiLSTM classifier using the Full feature vector.

Our first architecture used to determine the proper feature set was the SVM, which is most comparable to manual scoring approaches. This testing found that the DT/RMS feature set, using an SVM, produced inferior classification precision and a near-zero recall and F1_Micro_ score for REM (precision: 0.175; recall: 0.008; F1_Micro_: 0.0170) as compared with both wake (precision: 0.646; recall: 0.862; F1_Micro_: 0.738) and NREM (precision: 0.641; recall: 0.862; F1_Micro_: 0.738) in the mixed-treatment testing dataset. In addition, seizure state precision, recall, and *F*1_Micro_ scores were zero, and all postictal classification measures were zero. The Stat/RMS feature set improved wake (precision: 0.822; recall: 0.882; F1_Micro_: 0.851), NREM (precision: 0.784; recall: 0.773; F1_Micro_: 0.778), and REM classification (precision: 0.651; recall: 0.241; F1_Micro_: 0.352); however seizure and postictal both had unacceptable seizure (precision: 0.572) and postictal classification (precision: 0.183). The FFT/RMS feature set improved classification for the testing dataset in all states: wake (precision: 0.936; recall: 0.936; F1_Micro_: 0.936), NREM (precision: 0.897; recall: 0.911; F1_Micro_: 0.904), and REM (precision: 0.750; recall: 0.672; F1_Micro_: 0.709), seizure (precision: 0.516; recall: 0.276; F1_Micro_: 0.360), and postictal (precision: 0.318; recall: 0.401; F1_Micro_: 0.355). Finally, the Full feature set, when evaluated against the testing dataset, performed well for wake (F1_Micro_: 0.935) and NREM (F1_Micro_: 0.900), with lesser results for REM (F1_Micro_: 0.744), seizure (F1_Micro_: 0.825), and postictal (F1_Micro_: 0.426) classes. Overall, the SVM performed well for some states but is still below benchmark, even with improved performance with the Full feature set (Fig. 8).

### Multilayer architectures

We next compared four multilayer network architectures, as described above: dense layer, LSTM, BiLSTM, and Stacked-BiLSTM (Fig. 5). We compared these with 20 training epochs (passes through the training dataset, not to be confused with data epochs). The distribution of classification metrics varied substantially by class. Focusing on F1_Micro_, F1_Macro_, and F1_Weighted_ as well-rounded metrics described previously, we evaluated the F1 scores for all classifiers across our classes to determine the best-performing classifiers tested in our grid search. We evaluated the impact of feature vectors, classifier architecture, sequence length, and base layer unit size. After training, our results from ranking all parameters determined that the Full feature vector, BiLSTM architecture, seven-epoch input sequence, and 200-unit base layer size were the best-performing parameters in each respective category. This architecture, stopped at 20 epochs of initial training and achieved F1_Micro_ scores of 0.972 on wake, 0.957 on NREM, and 0.846 on REM, with an F1_Macro_ of 0.925 and F1_Weighted_ of 0.956 on the saline validation dataset. On the holdout testing dataset, this classifier achieved an F1_Micro_ on wake of 0.978, on NREM of 0.958, on REM of 0.887, on seizure of 0.782, and postictal of 0.741, with an F1_Macro_ of 0.869 and F1_Weighted_ of 0.965 (Fig. 8).

**Figure 9.**
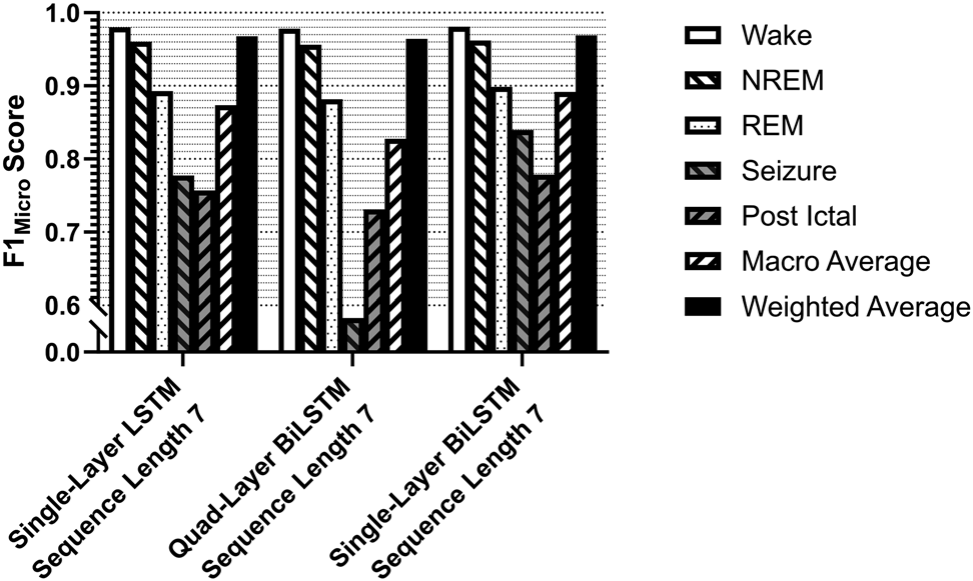
F1 metrics after complete training. F1_Micro_ scores for the individual states contained in the unseen and unlearned testing dataset (wake, NREM, REM, seizure, postictal) as well as F1_Macro_ and F1_Weighted_ were assessed for all of the LSTM-based variants trained with the optimized parameters: the Full 100-feature vector, an input sequence length of seven, and 200 base layer units. These three resulting architectures were trained to a limit of 60 epochs, with a loss patience of 0.001 over 5 epochs. Ultimately, the classifier that performed the best against the holdout real-world testing dataset was the single-layer BiLSTM, achieving an F1_Weighted_ of 0.968, an F1_Macro_ of 0.886, and an F1_Micro_ of the seizure state of 0.824.

While the BiLSTM performance was best, we next investigated somewhat similar performance of LSTM, BiLSTM, and the Stacked-BiLSTM architectures with increased training. All three of these classifiers were trained again with the seven-epoch input sequence, the 200-unit base layer size, and the Full feature vector from the beginning, this time with a training limit of 60 training epochs or to an early stopping threshold of 0.001, as defined in the Materials and Methods. F1_Micro_, F1_Macro_, and F1_Weighted_ metrics achieved by these models on the holdout testing dataset were used for the final evaluation. F1_Micro_ scores for the training, validation, and testing sets across all states, as well as F1_Weighted_ for these three models are ranked and presented in Figure 9.

**Figure 10.**
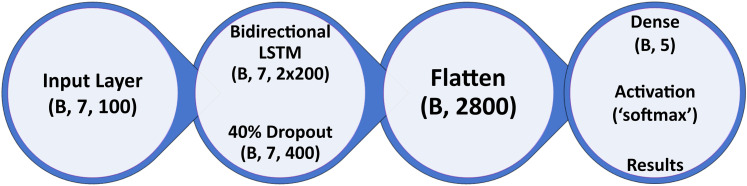
Final model architecture. The classification section of the model consists of an input layer for epoch sequences, followed by a BiLSTM layer of 200 units in each direction. The generalization of the classifier is improved by using an activity regularization function with an L1 of 0.0001 on the BiLSTM layer and then a 40% dropout layer. The output of this section is then flattened, and classification is performed by a five-unit dense layer with softmax activation.

Our final evaluation of these three classifiers found that a classifier with a seven-sequence-length input vector using the Full feature space with an architecture consisting of a 200-unit base layer single BiLSTM with a 40% dropout and a five-way softmax output layer was the most effective for classification of sleep as well as seizure and postictal state (Fig. 10, denoted as 7-Full-BiLSTM). This comprised our final product, the SWISC. F1_Micro_ scores for the SWISC for our validation dataset were 0.974 for wake, 0.959 for NREM, and 0.860 for REM, with an F1_Macro_ of 0.931 and an F1_Weighted_ of 0.959. In the case of the holdout testing dataset, F1_Micro_ scores for the SWISC were 0.981 for wake, 0.962 for NREM, 0.898 for REM, 0.840 for seizure, and 0.778 for postictal, with an F1_Macro_ of 0.891 and an F1_Weighted_ of 0.968. The final weighted accuracy across classes for this model was 96.59%. The mean FAR for seizure detection was 0.00745 seizures per hour in the testing dataset (*n* = 3 false alarms), 0 in the saline validation dataset, and 0.00217 for the training dataset (*n* = 3 false alarms), with a mean FAR of 0.00293 per hour over all of the manually scored files (*n* = 6 false alarms).

**Figure 11.**
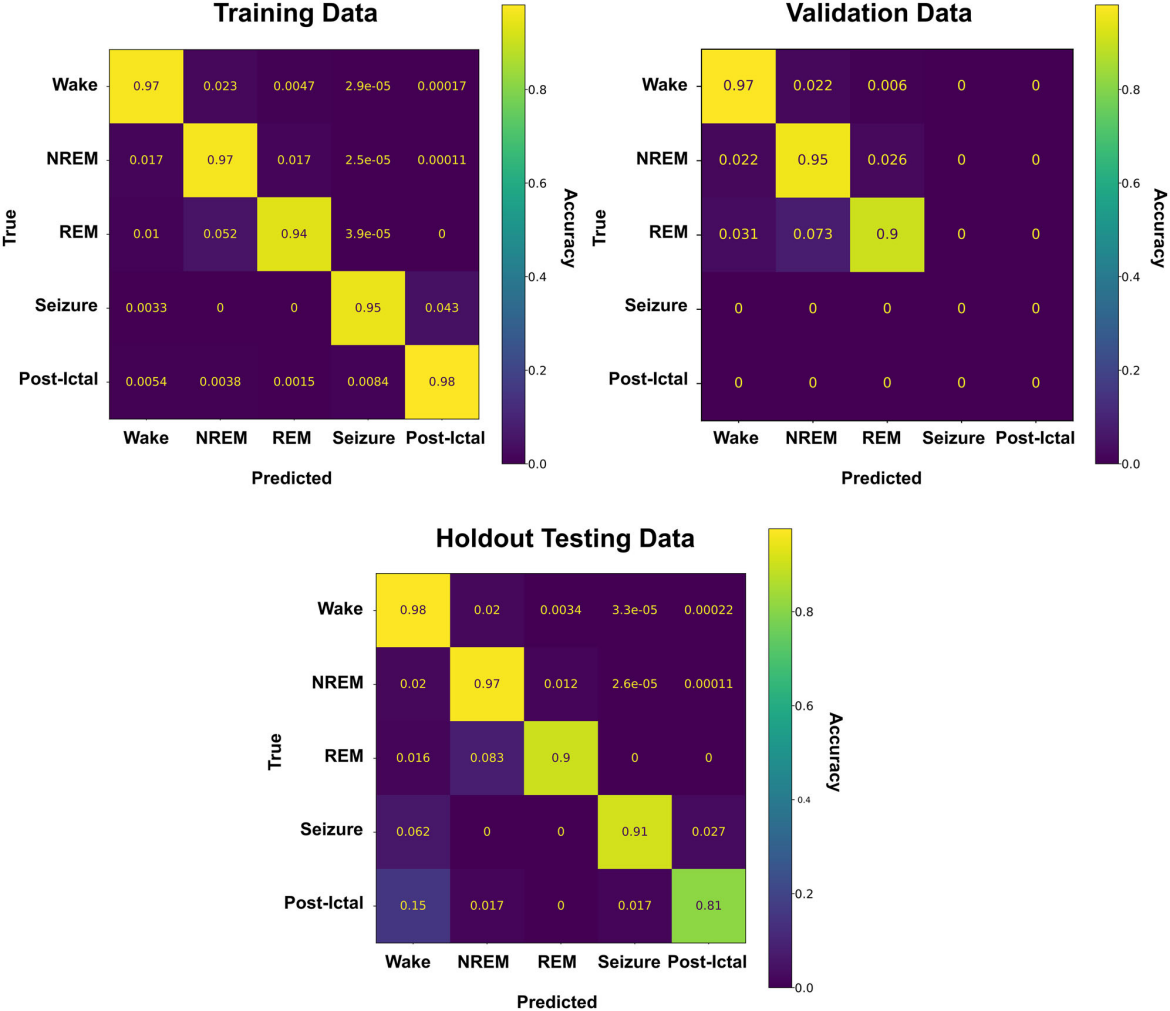
Performance after 60 training epochs. With the 2 × 200-unit initial layer size, the testing dataset was scored accurately compared with expert scoring (low 90%), mirroring its performance on the training and control validation datasets without overfitting.

**Figure 12.**
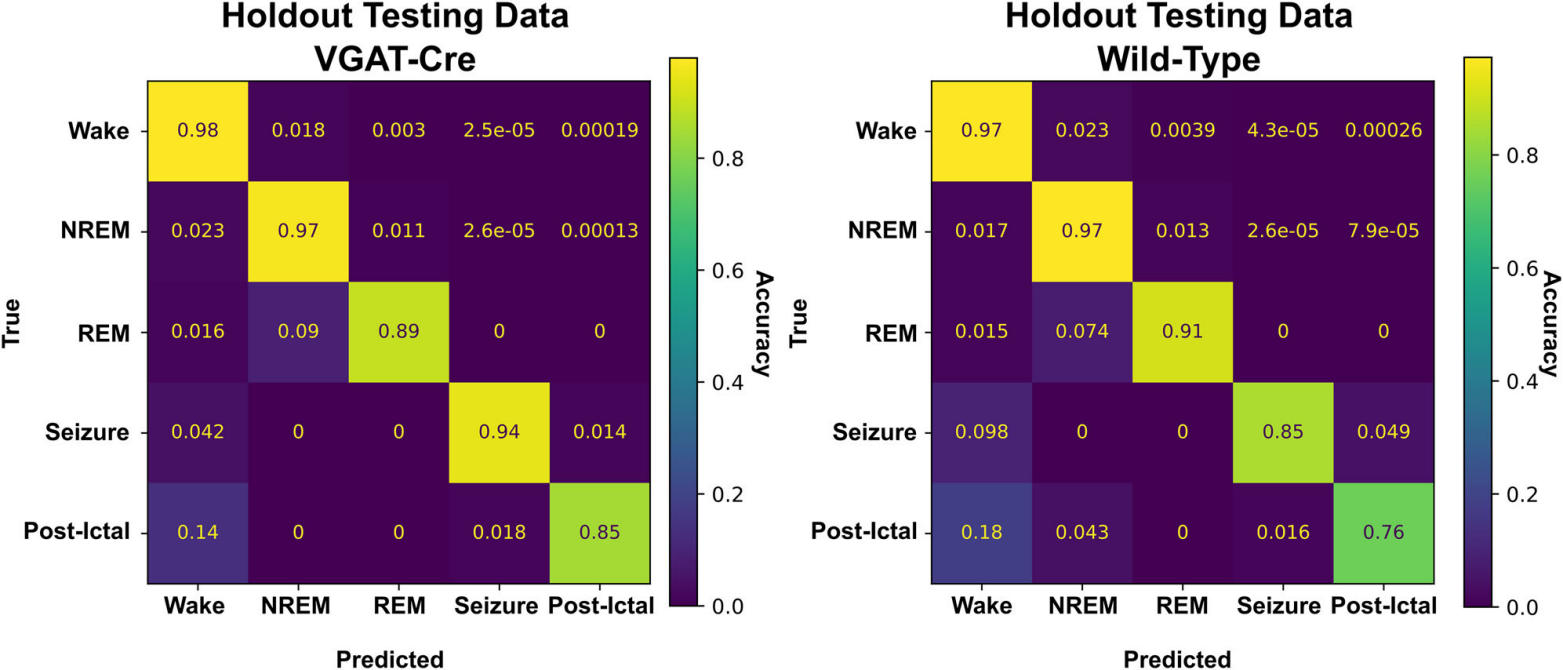
Performance on testing dataset by genotype. When the testing dataset is split by genotype into VGAT-Cre (*n* = 10, IAKA) and wild-type (*n* = 10; *n* = 7 IAKA) groups, performance is comparable between both genotypes. The only large difference between the per-stage precision values in these groups is in the classification of seizure, with seizure precision being only 0.85 in the wild-type dataset.

Confusion matrices produced for all training/validation/testing sets and all classes for the fully trained SWISC are presented in Figure 11, showing the true classification of wake, NREM, REM, and seizure at or above 90%, with variation in the postictal state accounted for its more qualitatively defined nature described earlier in this manuscript. Additionally, division of the testing dataset by genotype yields comparable results for both VGAT-Cre and wild-type animals, with the notable differences being seizure and postictal precision are 0.09 lower in wild-type animals versus the VGAT-Cre animals. Confusion matrices for each genotype in the testing dataset are shown in Figure 12. The complete breakdowns of all metrics for all models tested can be found in Extended Data 2.

### Channel dropping and applicability to other recording configurations

To better understand the individual electrophysiological channels’ contribution to the chosen architecture's scoring accuracy, we systematically removed individual channels. Then we trained new instances of the SWISC model out to 60 epochs or early stopping to properly evaluate these performances against the testing dataset.

Scoring based on hippocampal channels alone was highly effective. Training this architecture with solely our bilateral hippocampal channels, using all 20 statistical and spectral features present for each channel, produced F1_Micro_ scores on our testing dataset for wake at 0.964, NREM at 0.952, REM at 0.866, seizure at 0.818, and postictal at 0.872, with an F1_Macro_ of 0.914 and F1_Weighted_ of 0.949. This shows that even with half of the original classifier's inputs, robust classification is possible using this architecture. Adding EMG statistical and spectral features and RMS EMG to the dual-hippocampal montage produced similar F1_Micro_ scores, with wake at 0.975, NREM at 0.959, REM at 0.888, seizure at 0.833, and postictal at 0.882. F1_Macro_ and F1_Weighted_ in this condition were 0.923 and 0.962, respectively. It is also notable that when trained on the left hippocampal channel alone (the side of kainate injection), the classifier achieves F1_Micro_ results in wake of 0.962, NREM of 0.952, REM of 0.817, seizure of 0.841, and postictal of 0.813. F1_Macro_ and F1_Weighted_ in this condition were 0.860 and 0.947.

Scoring based on the input of the ECoG channel alone retained useful classification. F1_Micro_ scores in the testing dataset of this variant still reached usable levels with wake at 0.966, NREM at 0.941, REM at 0.757, seizure at 0.829, and postictal at 0.779. F1_Macro_ and F1_Weighted_ here were 0.840 and 0.945, respectively. The saline validation dataset in this variant achieved sleep classification F1_Micro_ scores of wake at 0.905, NREM at 0.931, and REM at 0.918, showing that the expansion of this classifier to sleep studies in much simpler montages in nonepileptic mice is achievable, broadening the potential reach of this classifier even further.

The addition of the EMG channel’s spectral and RMS features to the ECoG-only feature vector did not improve classification, in fact reducing the F1_Micro_ of seizure to 0.786 and postictal to 0.542 for the testing dataset.

**Figure 13.**
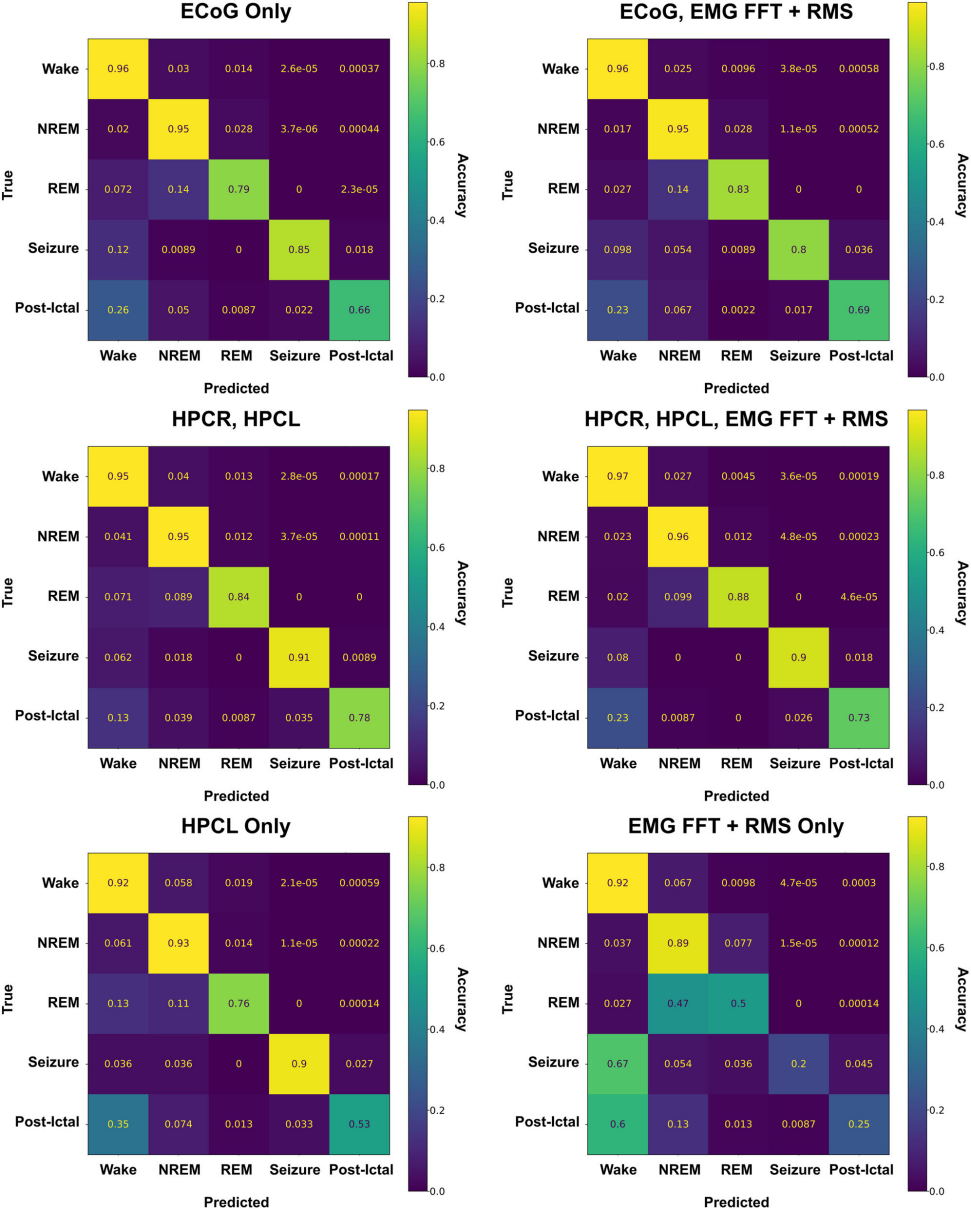
Interpretable machine learning via masking. Masking specific data channels during training, a hands-on method of interpretable machine learning which allows for the application of the classifier to recording configurations with less instrumentation, showed reliable classification with the SWISC model when used in any configuration that included a hippocampal channel. Classification accuracy did not drop significantly unless classification was performed without hippocampal signal or ECoG. This demonstrates that the classifier shows promise for various recording montages.

As expected, scoring on EMG spectral and RMS features alone poorly discriminated between forebrain-related states. This version of the classifier still achieved high F1_Micro_ scores for wake of 0.946 and NREM of 0.856 but faltered for REM with an F1_Micro_ of 0.484. Seizure had an F1_Micro_ of 0.271, and the F1_Micro_ of the postictal state was 0.308. While EMG could crudely classify sleep and wake states, it displayed poor performance with REM, seizure, and the postictal state. Confusion matrices for the testing dataset for all of these interpretable machine learning variants are displayed in Figure 13.

### Scoring results comparison

**Figure 14.**
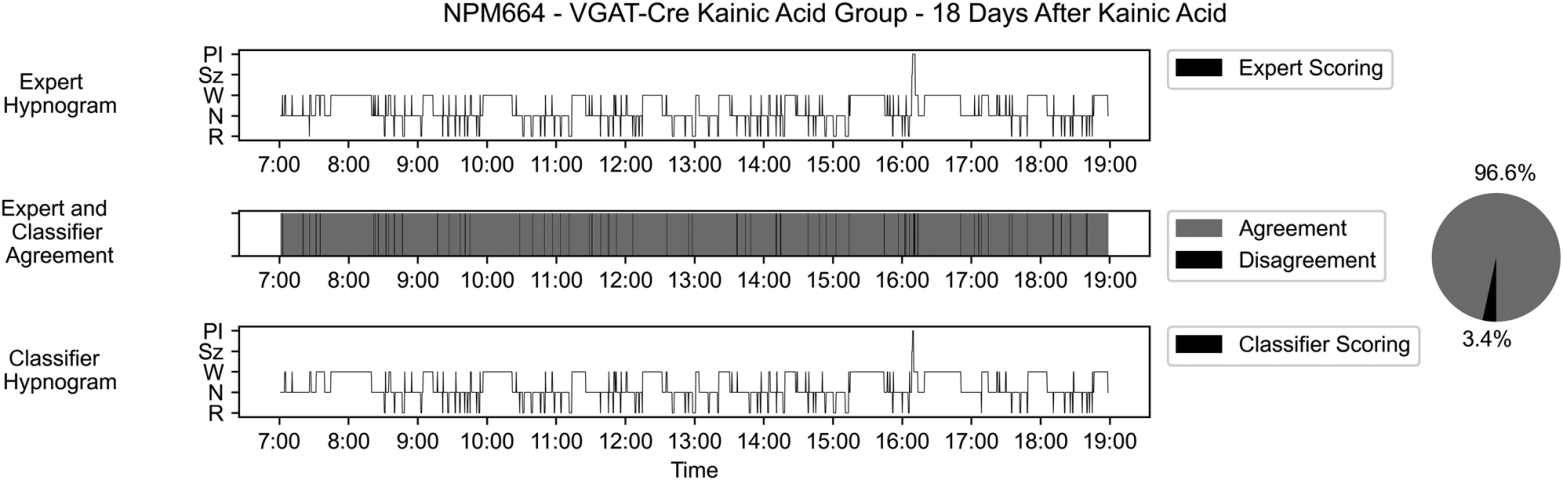
Scoring comparisons. To test the classifier, we chose to visually and computationally compare the agreement between expert scorers and the classifier for a representative 12 h record of sleep–wake. In an IAKA-treated animal, the classifier performs with ∼96% overall accuracy relative to expert scoring, which is comparable to inter-rater reliability on sleep–wake scoring tasks as demonstrated in Kloefkorn et al. (2020), where 93% agreement was reported between three expert scorers. Hypnogram legend for states: PI, postictal; Sz, seizure; W, wake; N, NREM; R, REM.

When ground-truth scores from manually scored components of the testing dataset are compared with those produced by the SWISC and rated for agreement across time epochs, average classification agreement with our ground-truth expert scores for epileptic mice in the holdout testing dataset is 96.41% (standard deviation ± 3.80%) when all states are accounted for. The Cohen's *κ* between manual and SWISC scoring for the entire testing dataset is 0.941. Holdout saline animals show an average agreement of 97.77% (standard deviation ± 1.40%). The average agreement across our full dataset of epileptic mice is 96.76% (standard deviation ± 3.30%). Additionally, there is a 96.38% (standard deviation ± 3.91%) agreement between ground-truth and classifier scores across recordings from saline-treated mice regardless of dataset. When the testing dataset is divided by genotype, agreement is 93.06% (standard deviation ± 4.85%) for wild-type IAKA mice, 95.47% (standard deviation ± 1.62%) for wild-type saline mice, and 93.05% (standard deviation ± 3.52%) for VGAT-Cre IAKA mice. Figure 14 contains a graphical representation of agreement and the corresponding hypnogram from expert scores and the classifier for an individual file to support the classifier's accuracy visually.

Scoring results from the SWISC were also evaluated according to Rechtschaffen and Kales criteria regarding state transitions (see Materials and Methods, Manual sleep and seizure scoring). The evaluation of our automatic scoring for these two rules found only 0.20% of the total epochs scored consisted of violations of these rules, with 26.55% of these violations being lone epochs of NREM and 73.44% being REM transition violations. This suggests that the classifier may have learned to implement some form of the Rechtschaffen and Kales rules. The Rechtschaffen and Kales layer is therefore provided, as it was in another paper from this lab (Exarchos et al., 2020) to correct any such violations.

### Performance on shorter epochs

A key final test for ensuring that our classifier is generalizable to other sleep analysis paradigms is ensuring that it works with differing epoch lengths. To this end, the files from our dataset were preprocessed with an epoch length of 4 s, as described in the preprocessing and feature extraction subsections. These differences in preprocessing and extraction were performed to test the limits of the classifier's accuracy. Average agreement for 4 s epochs was first assessed via comparison to the subepoched 20 s epoch scores, to ensure that no gross accuracy errors were introduced by reducing the temporal information used as input to the classifier. As the seven epochs of sequence input equate to a total of 140 s worth of features, this reduction in epoch length corresponds to the use of 28 s worth of features for 4 s epochs. We find that, across the entire testing dataset, there is an agreement between the subepoched 20 s scores and the 4 second classifier scores for animals in the IAKA group of 93.34% (standard deviation ± 4.13%) and for animals in the saline group of 95.47% (standard deviation ± 1.62%).

To further verify this 4 s scoring, we chose a subset of animals representing all combinations of genotype and condition group to manually score. The animals selected for this subsample were one VGAT-Cre saline animal from the validation dataset, one C57BL/6J wild-type saline animal, and one C57BL/6J wild-type IAKA animal, both from the holdout testing dataset, and two VGAT-Cre IAKA animals from the holdout testing dataset. A file each from 1 d before and 13 d after the delivery of IAKA or saline were used. The selection of using either dark or light files were made on a per animal basis, with the end result being a mix of day and night files for each cross-tabulation of genotype and condition. Though 10 files may seem small, this represents a sample of 108,000 manually scored epochs used for the 4 s validation and includes animals with electrographic artifacts.

All files were scored by BJH and then separately blindly scored by DJL. Inter-rater agreement for 4 s manual scores was 96.63% (standard deviation ± 2.95%). The agreement of these scores with the 4 s classifier scoring was 93.12% (standard deviation ± 4.41%) for BJH and 94.04% (standard deviation ± 3.87%) for DJL. The 4 s scoring also largely respects the Rechtschaffen and Kales criteria, with a violation rate of 0.64%, with 50.1% being lone NREM epochs and 49.9% of these being wake–REM transitions.

## Discussion

We present the successful creation of a machine learning-based classifier for the automated, accurate, and rapid scoring of sleep–wake states, seizures, and postictal states in mice with epilepsy. While previously reported classifiers effectively classified sleep in various populations of mice (Lampert et al., 2015; Yamabe et al., 2019; Exarchos et al., 2020; Grieger et al., 2021), none have demonstrated scoring proficiency in any rodent models of epilepsy. We also showed that even a highly effective sleep classifier (AccuSleep) does not perform well even when retrained to classify sleep–wake in training data from epileptic mice. The fairest comparison point for the performance of our retrained AccuSleep classifier is our SWISC ECoG/EMG submodel, as these are the same channels used in AccuSleep. This reduced version of our model outperforms AccuSleep in precision for all classes except REM. As demonstrated by this analysis and our grid search, our BiLSTM architecture empirically outperforms an existing classifier, SVMs, LSTMs, and dense neural net classifiers trained on the exact same data.

To our knowledge, our classifier is the first to achieve the goal of combined sleep–wake and seizure classification in mice with a variable phenotype from control to severe epilepsy. This thereby overcomes the infeasibility of comprehensive sleep–wake classification in epileptic mice that limited study of the important bidirectional interactions of sleep and epilepsy (Bernard et al., 2023; Sheybani et al., 2025). This classifier may have broad applicability given that classification performance remains high without EMG (common in studies of epilepsy) and even with ECoG or hippocampal LFP alone. However, we were not in a position to test the classifier with other epilepsy models that may have markedly different electrophysiological features, such as absence models. Models such as these with markedly different electrophysiologic phenotypes seem likely to require retraining of the classifier. An additional limitation of this implementation of the classifier includes the lack of a thorough feature-dropping assay. While we performed a channel-dropping assay to assess applicability to recording montages with fewer inputs, a full feature-dropping assay to create a leaner model was out of scope for our goal of creating a working sleep–wake and seizure classifier based upon expert-informed feature sets. It is possible that through feature-dropping analysis, a faster model could be made using fewer features, but in our case, the existing time savings even with our large model were more than adequate.

With our classifier, scoring time can be reduced to <3 min per 12 h recording file, including all preprocessing steps. There is no human input or time needed other than visual and statistical assessment of the results. These time savings will make the analysis of larger datasets feasible, as is often required in epilepsy studies due to the large individual variations in epilepsy severity. The time savings are also not traded off for any loss of precision in the sleep scores. The scoring accuracies of the classifier, with a weighted average of >95% for 20 s epoch lengths, exceed the 93% agreement in human scores (Kloefkorn et al., 2020) for AASM/Rechtshaffen and Kales sleep scoring between scorers in our lab, making it a well-rounded classifier for sleep and equivalent to a trained human scorer. The classifier's accuracy with respect to seizures is also apparent, with only six total falsely detected seizures, evenly split between the training and testing datasets. No false detections were discovered in the saline validation dataset. While all of these false detections were from the wake state, the low number of false detections supports the use of this classifier for combined sleep and epilepsy research.

Additionally, the automatic and manual validation of scoring at the 4 s epoch length, with an agreement of 93% to manually scored 4 s epochs, demonstrates the flexibility of this classifier to score at differing timescales without architectural or training adjustment. This point is particularly interesting given the reduction in temporal context provided to the BiLSTM via the input sequence of feature-extracted epochs. The input sequence, when data is processed in 20 s epochs, comprises 140 s worth of features, whereas for the 4 s epochs this is only 28 total seconds worth of data. Given that the 20 s trained model has been validated to accurately classify the data even when it is provided with less temporal context, this suggests that the model has learned some spectral features or interactions between features which are invariant between these two timescales. Further testing in this vein is a very intriguing future direction and could potentially provide insight into the temporal dynamics of sleep stage transitions.

The network architecture chosen fits well with the design intent. The innovation of BiLSTM is that it adds further information to the classifier by adding prior and future epochs in time series analysis rather than classifying the epoch at hand without sequence information (Graves and Schmidhuber, 2005). This likely accounts for the implicit inclusion of Rechtschaffen and Kales scoring rules behavior. On the other hand, the need for past and future epochs limits the use of the classified for immediate closed-loop control.

In summary, this classifier provides a rapid, accurate, robust, multifeatured sleep–wake and seizure scoring platform to those with access to basic computing resources. This tool will benefit the epilepsy research community as we conduct studies to better characterize and investigate the relationship between sleep, epilepsy, and other comorbidities.

## Data Availability

In order to enable the use of the fully trained models described in this manuscript, all Jupyter Notebooks used for preprocessing and training have been uploaded to GitHub (https://github.com/epilepsylab/SWISC) and are available as Extended Data 3, along with example files for testing importation and viewing file formatting specifications. The full dataset used for the training, validation, and testing splits is available upon reasonable request.

10.1523/ENEURO.0226-25.2025.d1 Data 1 Animal Exclusion, Group, and Recording Information. We have provided an Excel workbook with two sheets. “Animal Information and Exclusion” contains subject-specific information such as days of data recorded, days of data scored, genotype, IAKA or saline group, dataset assignment, mortality information, and exclusion criteria. “Inclusion and Exclusion Summary” contains simple counts per-genotype and per-condition for each dataset and exclusion criterion. Download Data 1, ZIP file.

10.1523/ENEURO.0226-25.2025.d2 Data 2 Metrics from All Models. This Excel workbook contains the imported metric values from the sklearn classification matrix generated for each model and each dataset, as well as pivot tables and prototype graphs used to determine which model is the most effective classifier. Download Data 2, ZIP file.

10.1523/ENEURO.0226-25.2025.d3 Data 3 Classifier Code. The root directory includes the SWISC_1.5.yml file for importing the classifier via Anaconda, and the folders “SWISC v1.5” and “Replication Code”. The files inside the SWISC v1.5 folder are identical to the files found on https://github.com/epilepsylab/SWISC. The Replication Code folder contains the exact script files and Jupyter Notebook used to train the models described herein, with all training history logged. Download Data 3, ZIP file.

## Synthesis

Reviewing Editor: Viji Santhakumar, University of California Riverside Decisions are customarily a result of the Reviewing Editor and the peer reviewers coming together and discussing their recommendations until a consensus is reached. When revisions are invited, a fact-based synthesis statement explaining their decision and outlining what is needed to prepare a revision will be listed below. The following reviewer(s) agreed to reveal their identity: Miriam Guendelman, Mathew Jones.

This paper describes the development of an algorithmic pipeline (architecture) for simultaneously scoring sleep states and epileptiform activity in mice, from EEG/EMG and hippocampal depth recording. The authors use a substantial data set consisting of 3,770 kainate (KA) and 650 baseline/control 12 h files, of which 1,176 (≈ 27 %) were manually annotated to compare several algorithmic approaches (e.g., SVM, CNNs, LSTMs and variants). They test these against human scoring of sleep and epileptiform states. They use standard metrics from signal detection theory for comparison. They also attempt to examine which data features are being used by the various algorithms. They find that both the pre trained and re trained AccuSleep CNN underperform, while a linear SVM fails to capture epileptic classes. Ablation studies indicate that SWISC remains robust even when limited to bilateral hippocampal LFPs or to a single cortical ECoG lead. They conclude that the novel bidirectional LSTM (BiLSTM) architecture called the Sleep-Wake and Ictal State Classifier (SWISC) outperforms others in being able to classify both sleep and epileptiform events. Strengths of the study include large, heterogeneous dataset across two genotypes, subject wise split plus class weighting to address imbalance and transparent, exhaustive hyper parameter search with open Jupyter notebooks and released weights. The authors describe the various architectures well, explain how they implemented and compared them, and provide extensive methods and code. The results for SWISC are impressive compared to other approaches. The authors have made major changes in response to previous critique. Overall, this is a thorough manuscript which addresses an important need in the epilepsy/sleep fields. The SWISC tool could be very beneficial to studies of sleep in epilepsy models.

The following remaining concerns need to be addressed.

1. In many cases, algorithms are "black boxes": they seem to work, but it's not easy to figure out how/why, or to figure out what "features" they care about. The authors addressed this problem by a) dividing input sets into "feature groups" based on their knowledge and intuition about sleep/epilepsy and b) examining the influence of dropping certain recording channels.

a) Input Sets are a useful way of breaking down the problem. But it depends entirely on author-preconceived notions about what composes a relevant "feature set". The authors have a few relevant sets of features in mind and they tested those with good results. What this shows is that the algorithm agrees that some features that the authors prefer are also used by the algorithm. However, it is unclear if it shows that these are the preferred features that the algorithm is using to make its decision. Please comment on/discuss this issue.

b) Dropping Channels - Channel dropping is a useful experiment for practical reasons. But theoretically it's not obvious how "channel" relates to "feature". How does dropping a channel impact the "feature" that is used for classification? Please comment on the utility of channel dropping as a viable test for features in a multidimensional space. The channel is only one dimension, and maybe not a dimension of particular interest by itself. Please clarify how Channel dropping was implemented and interpreted.

The concern with the channel dropping assay relates to what "features" versus "channels" contribute to classification. 1 ) Each channel is a continuous stream of data that *contains* certain "features", possibly signifying an event of interest. The channel by itself is not a "feature" of interest. 2) A "feature" can be an *event* that is spread across multiple channels. For example, the *combination* of something on certain EEG channels with something on certain EMG channels when trying to decipher sleep states. It's the cross-channel information that's the relevant "feature", not any individual channel. If one drops the EMG channel, then one is not left with one less "feature", but rather a degraded "feature" that's missing a certain dimension. So, channels and features are the same thing in a multidimensional data space as described in this paper. While the authors are not directly equating features and channels, some clarification in the context of the channel dropping studies would be helpful.

2. Methods: Line 80 - It seems that VGAT-Cre mice were more susceptible to death or tech failure than WT? What about susceptibility to epilepsy? Or baseline sleep disorders compared to WT? Is the Cre interfering with normal processing of VGAT protein? Please comment.

3. Dataset description &composition: Please provide a per animal summary supplementary table detailing total hours recorded, hours manually scored, and the train/val/test allocation and portion of each label. This will clarify data balance and facilitate future reuse. Excluded animals (L 80) - report recording length for each and justify why partial data were not incorporated, especially for epileptic mice. Line 108 - Please provide sample rate &filtering for the different EEG/EMG channels. Please provide video frame rate.

4. Line 114 - Was video and EEG/EMG recorded prior to, during and after KA infusion? If so, there is useful data about the severity of "initial insult" and how initial insult severity impacts future sleep/epilepsy trajectory. Please comment.

5. Manual scoring &label resolution: In some parts it is clear where multiple humans scored the same data set, but not always. Please clarify throughout. Also, in a few cases, performance of an algorithm/architecture is compared with inter-human comparisons. This is an extremely useful comparison and should be expanded throughout. At discretion of the authors.

Inter scorer agreement (L 61-63) - Line 61-63 "The agreement between scorers in our laboratory is high at > 93%, [Citation removed for anonymization], in accordance with..." it is not clear if this is for the scoring of sleep / seizure or both, what is the metric ( accuracy, or kappa or another agreement metric) - please clarify. Also, in human scoring agreement between human scorers is lower (with kappa values around 0.75-0.8), making the prior statement out of place - see: sleep - https://pubmed.ncbi.nlm.nih.gov/37309714/

seizures - https://pubmed.ncbi.nlm.nih.gov/32472781/

6. Pre processing &feature extraction: Was the filtering performed per file or per epoch, please clarify.

Do the 12-hour files include a balanced number of hours in lights on and lights off data? if not discuss how this may affect scaling by file when including different proportion of sleep/wake.

7. The authors use both welch and FFT to extract power band information, this seems a bit redundant considering the short epochs for feature estimation, how correlated are the resulting features by the two approaches? and could a model train on bands extracted with a single approach can yield a similar performing leaner model?

8. Figure 2 should include more detail on each step rather than file sizes (the number of samples I think may be more relevant), for example in the feature extraction this should list the features groups extracted per electrode, and/ or the different normalization steps (band width and broadband power) for example.

9. Model training &validation: it is strange that only saline control animals were used for validation and loss monitoring, this will allow to track convergence on sleep related labels but not seizures. Inherently this is not a problem as algorithmic performance is finally evaluated on the test which includes representation of all groups, this choice should be better explained.

- Line 231 - "Next, a linear support vector machine", emphasize that LinearSVC was used for this separation. Also was this classifier trained with class wights to account for imbalance? If not, this could explain line 394 stating "seizure state precision, recall, and F1Micro scores were zero, and all post-ictal classification measures were zero" and the low performance in these classes with other feature variations.

- For LSTM with input size 1, the author should explain that this is a baseline condition used to quantify the value of temporal context.

- Only 10 files from 5 animals were used for the performance evaluation of the 4 seconds, how were these files elected? it is not clear to me how this evaluation was performed? Was the best model for 20 seconds used? in such case 7 epoch sequences previously derived from 140 seconds of data now include only 28 seconds, I see how this can technically be done but shouldn't this affect the temporal context of the LSTM and the distinction of states in the data?

10. Benchmark against AccuSleep

- re-trained only on 109 files and evaluated on a subset of test files. For better benchmarking the similar training, validation and test should be used for this analysis, or performance should be compared to the performance in these files for other approaches.

- The model was retrained on 4 classes wake, NREM, REM, and unknown, not like the evaluated in-house classifier making the performance metrics incomparable. Further, the author states that no seizure epoch were classified as "unknown" however from the confusion matrix presented in figure 4 no epochs of any class are classified as unknown and practically only assigned to wake/ nrem /rem categories, could the authors explain how this happens?

- AccuSleep uses 2 EEG electrode and EMG and should be compared to a model trained on similar data.

- Section 3.1 - the authors hypothesis that the existing classifiers will underperform in epileptic mice. Did the AccuSleep perform better in the non-epileptic mice compared to the epileptic, or is the performance drop just due to a data drift, this is an important comparison to support this claim?

- Lines 502-506 - "We also showed that even a highly effective sleep classifier (AccuSleep) does not perform well even when re-trained to classify sleep-wake in training data from epileptic mice...." - this claim is not sufficiently supported by the current analysis due to the fact that retraining was performed only on part of the data the BiLSTM was trained on and the same for evaluation, moreover there is the point of the "unknown" class that needs to be accounted for

11. Performance reporting:

-Beyond the metrics presented in the paper, in seizure detection due to the unbalanced nature of the problem, it is ill reflected in measures such as F1, thus it is acceptable to report the false alarm rate per hour along with the sensitivity, to evaluate the practical value of the classifier in this task.

- Further, in sleep scoring agreement is usually quantified by both kappa and F1 for only sleep stages, thus for comparison with prior sleep scoring benchmarks it may be useful to evaluate performance on interictal epoch separately.

- How do performance metrics change in the different animal groups?

- It is recommended that the authors create a summary table comparing the key performance metrics of the models tested, making it easier to compare the performance.

- confusion matrices in figure 8 for the validation data should include predicted seizure and postictal to account for false detections if they occur or show their absence

12. Only ten files from five animals were analyzed for the 4 second evaluation experiment. Clarify selection criteria and discuss the reduced temporal context (7 × 4 s ≈ 28 s) relative to the 20 s model (≈ 140 s).

-

Minor comments

- Line 50, in the rebuttal the authors state that they replaced the use of the term "parametric" but it is still used here

- Line 130-131 - "and high theta activity, particularly in hippocampal electrodes, with the latter becoming less prominent in some epileptic mice" - has this been previously described in the literature? if so, please provide a citation

- Line 292 - "where layer size is denoted in figures and tables" - what figures and tables?

- Figure 3 - missing reference in Methods 2.11.3.

- Figure 7 - redundant; consider merging its information into Figure 3.

- Figure 10 - could be moved to Supplementary Material.

- Abbreviations (RMS, BiLSTM, etc.) should be defined at first mention.

- Computational Resources - could the authors mention the minimal computational requirements to run inference on new data

## Author Response

Author's Rebuttal Dear eNeuro Editors and Reviewers, Thank you for your insightful and constructive feedback on our manuscript - we think that the paper has been improved based on the reviewers' suggestions. Overall, we think that we have been able to address all comments. Responses are in-line with reviewers' comments, which are in bold below. Thank you again for considering our manuscript.

Comment:

1. In many cases, algorithms are "black boxes": they seem to work, but it's not easy to figure out how/why, or to figure out what "features" they care about. The authors addressed this problem by a) dividing input sets into "feature groups" based on their knowledge and intuition about sleep/epilepsy and b) examining the influence of dropping certain recording channels. a) Input Sets are a useful way of breaking down the problem. But it depends entirely on author-preconceived notions about what composes a relevant "feature set". The authors have a few relevant sets of features in mind and they tested those with good results. What this shows is that the algorithm agrees that some features that the authors prefer are also used by the algorithm. However, it is unclear if it shows that these are the preferred features that the algorithm is using to make its decision. Please comment on/discuss this issue.

Response:

Thank you for this comment. We agree that the grouping of feature sets has some logic, but may not be in agreement with what is most critical for the classifier. A combinatorial approach could be taken to exploring which components of the feature sets are most important. Time taken for computation is less of a concern with contemporary resources. However, we still wanted to create an efficient algorithm that made use of conceptual feature sets that have an a priori logic or were based on prior work. Theta to delta ratio and EMG are typically the most helpful features, but we have shown that the addition of further feature sets improves classification. We could further explore the minimal feature set that would be effective - this is a substantial undertaking given the combinatorial nature of the problem. We have, for now, instead discussed this point in the manuscript:

Comment: b) Dropping Channels - Channel dropping is a useful experiment for practical reasons. But theoretically it's not obvious how "channel" relates to "feature". How does dropping a channel impact the "feature" that is used for classification? Please comment on the utility of channel dropping as a viable test for features in a multidimensional space. The channel is only one dimension, and maybe not a dimension of particular interest by itself. Please clarify how Channel dropping was implemented and interpreted.

The concern with the channel dropping assay relates to what "features" versus "channels" contribute to classification.

1 ) Each channel is a continuous stream of data that *contains* certain "features", possibly signifying an event of interest. The channel by itself is not a "feature" of interest.

2) A "feature" can be an *event* that is spread across multiple channels. For example, the *combination* of something on certain EEG channels with something on certain EMG channels when trying to decipher sleep states. It's the cross-channel information that's the relevant "feature", not any individual channel. If one drops the EMG channel, then one is not left with one less "feature", but rather a degraded "feature" that's missing a certain dimension. So, channels and features are the same thing in a multidimensional data space as described in this paper. While the authors are not directly equating features and channels, some clarification in the context of the channel dropping studies would be helpful.

Response:

Thank you for this point. We agree that there is far from a one-to-one correspondence between features and channels. We now realize that our description of this was misleading and confusing. The overall goal of this section of the paper is to explore whether the classifier could work on quite distinct datasets that have different data elements or montages. In epilepsy research, recording EMG is less common, and not all labs make direct microelectrode recordings from the hippocampus. We hoped to show that this classifier is likely to be helpful in these settings, even without EMG and a typical sleep-wake rodent recording montage (frontal and contralateral parietal). There is some correspondence between features and channels (e.g., EMG, or theta in hippocampus, or good theta and delta recordings with the frontal-contralateral parietal montage), and this had led us to erroneously describe this as about features. Interpretability may still be somewhat applicable, given insight into which channels are most essential for classification, but this has also been modified in the text to better describe the objective of channel dropping.

Comment:

2. Methods: Line 80 - It seems that VGAT-Cre mice were more susceptible to death or tech failure than WT? What about susceptibility to epilepsy? Or baseline sleep disorders compared to WT? Is the Cre interfering with normal processing of VGAT protein? Please comment.

Response:

Thank you for this observation. We reviewed these findings in detail. Our initial reporting of the death rates of VGAT-Cre and wild-type mice was in error due to the marking of 3 wild-type animals, which had both signal errors and early death, being excluded for signal error alone. This has been corrected (lines 79-81), showing our similar mortality rate between VGAT-Cre and wild-type animals.

Homozygous VGAT-Cre mice have been shown to have reduced VGAT mRNA and protein levels, and have been found to have altered GABAergic transmission in the hippocampus, and develop spontaneous recurrent seizures after electrical kindling, suggesting that the transgene is pro-epileptogenic (Straub, et al. 2020). Despite this, we have studied these mice extensively and have never recorded spontaneous seizures, and mice, in our hands, and Jackson Laboratories, the homozygous mice have a normal phenotype (see https://www.jax.org/strain/028862#). We have not found a difference between wild-type and VGAT-Cre mice, with the burden of seizures, seizure severity (Racine score), seizure duration, or mortality, but we may be underpowered to detect small differences. Given the normal phenotype, lack of spontaneous seizures, and variable susceptibility of strains (and even sub-colonies) of mice to epileptogenesis (e.g., Bankstahl et al., 2012), we have not further pursued a direct comparison of strains or heterozygous versus homozygous mice. We have now mentioned this in the manuscript in Section 3.2 lines 396-403.

Comment:

3. Dataset description &composition: Please provide a per animal summary supplementary table detailing total hours recorded, hours manually scored, and the train/val/test allocation and portion of each label. This will clarify data balance and facilitate future reuse.

Response:

Thank you. This is now provided as Extended Data 1.

Comment:

Excluded animals (L 80) - report recording length for each and justify why partial data were not incorporated, especially for epileptic mice.

Response:

As stated above, we have included animal-specific file count information for all animals, as well as details on why a given mouse was excluded in Extended Data 1. Some partial data were incorporated, unless the overall recording time was low (i.e., died soon after or during epileptogenesis). This is explained in more detail on lines 378-394 of the article file.

Comment:

Line 108 - Please provide sample rate &filtering for the different EEG/EMG channels. Please provide video frame rate.

Response:

This change has been addressed in lines 102-104 in the article file, and this information was included in the new version of Figure 2 outlining our pre-processing as well.

Comment:

Line 114 - Was video and EEG/EMG recorded prior to, during and after KA infusion? If so, there is useful data about the severity of "initial insult" and how initial insult severity impacts future sleep/epilepsy trajectory. Please comment.

Respone:

This is clarified on what are now lines 111-113 in the article file. This paper is principally methodological and focused on the classifier, so we do not address this and many other scientific questions in this manuscript.

Comment:

5. Manual scoring &label resolution: In some parts it is clear where multiple humans scored the same data set, but not always. Please clarify throughout. Also, in a few cases, performance of an algorithm/architecture is compared with inter-human comparisons. This is an extremely useful comparison and should be expanded throughout. At discretion of the authors.

Response:

Thank you very much for this comment. We neglected to phrase this section in a way that remained clear after the redaction of author initials. We have updated this section and made it much clearer when redacted (lines 118-120), confirming all scoring was performed by two independent reviewers. All manual scoring for four-second resolution was also scored by two independent reviewers and similarly clarified in lines 561-565 of the article file.

Comment:

Inter scorer agreement (L 61-63) - Line 61-63 "The agreement between scorers in our laboratory is high at > 93%, [Citation removed for anonymization], in accordance with..." it is not clear if this is for the scoring of sleep / seizure or both, what is the metric ( accuracy, or kappa or another agreement metric) - please clarify. Also, in human scoring agreement between human scorers is lower (with kappa values around 0.75-0.8), making the prior statement out of place - see: sleep - https://nam13.safelinks.protection.outlook.com/

Response:

We have clarified on lines 60-61 of the article file that this agreement, cited to previously published work, is related to scoring sleep only and is based on inter-rater accuracy. As these are values of mouse sleep scoring agreement, the human literature is less relevant to this point than the previous mouse-specific evidence (Kloefkorn et al., 2020, 2022) and others (Rytkönen, et al., 2011). The mouse recordings are intracranial, all mice have a similar epilepsy, and the electrodes are appropriately located, so the detection of heterogeneous seizures on artifact-prone and non-intracranial human scalp EEG is a very different scenario.

Comment:

6. Pre processing &feature extraction: Was the filtering performed per file or per epoch, please clarify.

Response:

Thank you. Filtering was performed per file, and feature extraction was performed per-epoch. This has been clarified on line 167-170 as well as in Figure 2 detailing the pre-processing.

Comment:

Do the 12-hour files include a balanced number of hours in lights on and lights off data? if not discuss how this may affect scaling by file when including different proportion of sleep/wake.

Response:

Excellent question, which is very relevant to the classifier's goal of cross-laboratory applicability. In our laboratory, the start of recording for our 12-hour files is triggered by a custom Arduino-based light and recording controller, and each file therefore either has lights on or off. While this may affect Z-scores, due to a relatively larger amount of increased high-amplitude NREM during the light phase, for example, our spectral normalization ensures that our primary features are those of relative power in frequency bands of interest. Only three features are affected by the Z-scoring: mean, median, and standard deviation. Skewness and kurtosis are scale-invariant, and all spectral features are normalized to broadband power regardless of initial signal scale. Additionally, we find no difference in comparison of classifier scores to manual scores relative to the light phase, so the impact of light-phase on scaling does not appear to impact classification.

Comment:

7. The authors use both welch and FFT to extract power band information, this seems a bit redundant considering the short epochs for feature estimation, how correlated are the resulting features by the two approaches? Response:

Thank you, this is a very valid concern from a signal-processing perspective. FFT and Welch were used together because, as mentioned on what is now lines 185-186, the formula for Welch PSD is also normalized relative to the width of the frequency bin. This provides slightly different values in our context, where our lower- and higher-frequency bins are of markedly different widths.

A correlation analysis of these features in the ECoG channel, across a joined array of the train, validation, and test datasets, show that FFT and PSD features in the Delta band have a Pearson's r of 0.910. The highest correlation is our 61-99 Hz band, r=0.94 between FFT and PSD, and the lowest is our high theta band, r=0.73. For the left and right hippocampi, these correlations are much lower, with the minimum in the 12-20 Hz band (HPCL: r=0.12, HPCR: r=0.15 ) and the maximum in the 30-55 Hz band (HPCL: r=0.72, HPCR: r=0.75). Overall, there is not complete redundancy and scoring performance is improved when both are used (see immediately below; taking into account considerations from Reviewer Comment #1 above).

Comment: and could a model train on bands extracted with a single approach can yield a similar performing leaner model? Response:

This was directly tested for via the comparisons of Feature Set 2, Feature Set 3 and Feature Set 4 (lines 197-204). Feature Set 2 determined if statistical features and RMS EMG were sufficient for scoring, Feature Set 3 determined if FFT and RMS EMG were sufficient, and neither performed as well as the feature set with the statistical features, the FFT, the PSD, and the RMS EMG. Additional overhead for extracting/scoring FFT or PSD vs FFT alone is negligible, so "leaner" is not highly prioritized in our design parameters. We are primarily motivated by scoring accuracy, with speed/memory savings being second.

Comment:

8. Figure 2 should include more detail on each step rather than file sizes (the number of samples I think may be more relevant), for example in the feature extraction this should list the features groups extracted per electrode, and/ or the different normalization steps (band width and broadband power) for example.

Response:

Thank you - the original figure was unclear. This change has been addressed, with sample sizes per-channel for each mouse in a file reported for the SMRX origin, and export to '.mat' files. Feature extraction details have been added to the appropriate step, with the subsampling of these features to make feature sets reserved for Figure 2-1 as before.

Comment:

9. Model training &validation: it is strange that only saline control animals were used for validation and loss monitoring, this will allow to track convergence on sleep related labels but not seizures. Inherently this is not a problem as algorithmic performance is finally evaluated on the test which includes representation of all groups, this choice should be better explained.

Response:

As our previous description was unclear, this has been updated in lines 216-217. This was deliberate given the need to track convergence of sleep scoring only and therefore only from non-epileptic mice.

Comment:

Line 231 - "Next, a linear support vector machine", emphasize that LinearSVC was used for this separation. Also was this classifier trained with class wights to account for imbalance? If not, this could explain line 394 stating "seizure state precision, recall, and F1Micro scores were zero, and all post-ictal classification measures were zero" and the low performance in these classes with other feature variations.

Response:

This is an excellent point that was not fully explained. We did train all classifiers with class weights. As line 231 and the associated section are intended to overview the classifier types tested, the addition of our explanation of the use of class weighting and the specific loss function used with the SciKitLearn's SGDClassifier function were added on lines 263-264 with the existing description of the SVM implementation. The total lack of seizure and post-ictal classification that was quoted was specifically the DT/RMS dataset, which contains essentially no data that could be used, even by a human, to discriminate seizure from wake.

Comment:

- For LSTM with input size 1, the author should explain that this is a baseline condition used to quantify the value of temporal context.

Response:

Thank you for this feedback - this was our intention for this condition, and it was not adequately explained. This explanation has been addressed on lines 298-299.

Comment:

- Only 10 files from 5 animals were used for the performance evaluation of the 4 seconds, how were these files elected? it is not clear to me how this evaluation was performed? Was the best model for 20 seconds used? in such case 7 epoch sequences previously derived from 140 seconds of data now include only 28 seconds, I see how this can technically be done but shouldn't this affect the temporal context of the LSTM and the distinction of states in the data? and 12. Only ten files from five animals were analyzed for the 4 second evaluation experiment. Clarify selection criteria and discuss the reduced temporal context (7 × 4 s ≈ 28 s) relative to the 20 s model (≈ 140 s).

Response:

This is a very helpful set of questions.

We have revised the file selection to include only animals from the validation (n=1) and testing (n=4) datasets. The selection of 10 12-hour files, at four seconds per epoch, means this manual validation on a total of 108,000 epochs, which should be more than reasonable for accuracy calculations.

Two files each were selected for manual scoring, with the first being from the final day of baseline and the second from 13 days post-IAKA or saline delivery. Additionally, for coverage of both lights-on and lights-off timing, we scored two lights-on files from VGAT-Cre saline, two lights-off files from C57BL/6J saline, two lights-on files from C57BL/6J IAKA, and two files from each light period for VGAT-Cre IAKA, ensuring all light, genotype, and condition combinations were validated.

There was no significant difference in inter-rater or manual/classifier agreement for any genotype, condition, time point, or lighting condition.

The four-second files were evaluated with the SWISC model, the same model we selected for performance on 20-second epochs. We have additionally included the comparison of the sub-epoched 20-second scores to the four-second classifier scores to show there are no gross scoring violations in the dataset at large (lines 547-552). The manual scoring validation, as a spot check, was performed by comparing overall epoch-by-epoch accuracy vs. each scorer, as well as assessing inter-rater accuracy on a per epoch basis.

While the reduction in context length for the LSTM is a relevant concern, our manual scoring has validated that in these animals our scoring is accurate regardless of the temporal context mechanics at play. We can only conclude, happily, that this change in temporal scale between conventionally used epoch lengths, did not markedly change epoch features and sequence-related classification. A short discussion of this point has been added to the Discussion section, lines 601-610.

Comment:

10. Benchmark against AccuSleep - re-trained only on 109 files and evaluated on a subset of test files. For better benchmarking the similar training, validation and test should be used for this analysis, or performance should be compared to the performance in these files for other approaches.- Response:

Thank you for these concerns about our analysis of AccuSleep performance. We have performed an extensive re-evaluation of AccuSleep, including validating it on the same testing dataset as the other classifiers. Additionally, for the re-trained 20-second version of AccuSleep, this was trained using our full training dataset in this iteration. This constitutes a fairer and more logical comparison and is now updated in the manuscript.

Comment:

The model was retrained on 4 classes wake, NREM, REM, and unknown, not like the evaluated in-house classifier making the performance metrics incomparable. Further, the author states that no seizure epoch were classified as "unknown" however from the confusion matrix presented in figure 4 no epochs of any class are classified as unknown and practically only assigned to wake/ nrem /rem categories, could the authors explain how this happens? Response:

Addressing this question has clarified our treatment of AccuSleep. The "unknown" state was not adequately explained: As a feature of its design, the 'unknown' state is not usable for classification by AccuSleep, as AccuSleep cannot classify any fourth class aside from Wake/NREM/REM. We used this state score to code, and aggregate, both 'seizure' and 'post-ictal' states for the purposes of validation against the baseline AccuSleep and our re-trained version, and the coding of our "seizure/post-ictal" category necessitated using the same integer code as "Unknown". This prevents AccuSleep from training on these epochs as if they were sleep-wake states and thus disturbing subsequent sleep-wake classification.

While one could argue AccuSleep's classification of our true merged Seizure/Post-Ictal group as Wake is, in a sense, a true classification, it regardless demonstrates that AccuSleep does not have the epilepsy-specific capabilities we were seeking to design and cannot be adapted to do so without significantly changing that program's design.

We believe this analysis is sufficient, as our purpose in employing AccSleep as a benchmark was merely to demonstrate that sleep stages are not classified in our epileptic animals accurately by existing works, not to attempt to validate whether AccuSleep could or could not be trained to evaluate a fourth and/or fifth class. In summary we believe this is the most effective benchmark for our problem, as there is no extant architecture which seeks to simultaneously classify sleep and seizure.

Comment:

AccuSleep uses 2 EEG electrode and EMG and should be compared to a model trained on similar data.

Response:

We agree, but the recording configuration used for the original AccuSleep paper is unconventional and less preferred for sleep-wake recording: Both electrodes are placed over the cortex overlying the dorsal hippocampus at the same mirrored lateral and A-P coordinates, without a bipolar frontal contralateral parietal montage; it is unclear from the paper, but review of the data in the Open Science Framework reveals that only one EEG channel was used and likely (again not stated) in referential configuration (comparison to screw over the cerebellum). We cannot make a direct comparison to this recording configuration. Nonetheless, we retrained AccuSleep with data from a recording configuration that is generally thought to be better for sleep-wake scoring. It did not perform as well as our classifier. We still addressed our hypothesis that existing sleep scoring classifiers would struggle with epileptic mice with sleep-wake scoring and would not handle seizure classification. We have discussed this question in the Discussion section on lines 575-579.

Comment:

- Section 3.1 - the authors hypothesis that the existing classifiers will underperform in epileptic mice. Did the AccuSleep perform better in the non-epileptic mice compared to the epileptic, or is the performance drop just due to a data drift, this is an important comparison to support this claim? - Lines 502-506 - "We also showed that even a highly effective sleep classifier (AccuSleep) does not perform well even when re-trained to classify sleep-wake in training data from epileptic mice...." - this claim is not sufficiently supported by the current analysis due to the fact that retraining was performed only on part of the data the BiLSTM was trained on and the same for evaluation, moreover there is the point of the "unknown" class that needs to be accounted for Response:

Thank you very much for these comments. We should have clarified this and have now added this to the manuscript. We have broken down AccuSleep's performance on the whole testing dataset into Saline and IAKA cohorts and presented this as Figure 4-1, where it is evident that the misclassification of sleep states is entirely dependent on the Saline vs. IAKA condition. Additionally, as mentioned above, we have updated all AccuSleep-based analyses using the exact same training and testing input files as the other classifiers, making these various models as comparable as possible.

Comment:

11. Performance reporting:

-Beyond the metrics presented in the paper, in seizure detection due to the unbalanced nature of the problem, it is ill reflected in measures such as F1, thus it is acceptable to report the false alarm rate per hour along with the sensitivity, to evaluate the practical value of the classifier in this task. - Further, in sleep scoring agreement is usually quantified by both kappa and F1 for only sleep stages, thus for comparison with prior sleep scoring benchmarks it may be useful to evaluate performance on interictal epoch separately.

Response:

From swift research (a PubMed search for "seizure AND classification and "F1 score"", F1 score is used in 68 papers as a primary metric of classification. As we have reported recall, which is calculated as True Positives/(True Positives+False Negatives), we have already reported sensitivity. While we agree that the use of F1 alone for sleep stages while including seizure and post-ictal epochs is atypical, these two classes make up only 0.1% of our extant data, making their impact on sleep scoring F1 negligible while making F1 a very useful metric for assessing those classes specifically. Cohen's kappa has been employed on the 20-second epoch classifier data now, and is reported on line 527.

Comment:

- How do performance metrics change in the different animal groups? Response:

Classification, as represented by the confusion matrix, is comparable between wild-type and VGAT-Cre testing dataset animals, save a small dip in seizure precision in the wild-type animals relative to VGAT-Cre. This is now represented as Figure 8-1.

Comment:

- It is recommended that the authors create a summary table comparing the key performance metrics of the models tested, making it easier to compare the performance.

Response:

This summary table is extremely large due to the number of models tested, but is now included as Extended Data 2.

Comment:

- confusion matrices in figure 8 for the validation data should include predicted seizure and postictal to account for false detections if they occur or show their absence Response:

Thank you very much for this observation. This confusion matrix has been updated to show all five labels.

We thank you kindly for your suggestions! Sincerely, Brandon Harvey &Nigel Pedersen On Behalf of the Authors
